# Isomorphic Insertion of Ce(III)/Ce(IV) Centers into Layered Double Hydroxide as a Heterogeneous Multifunctional Catalyst for Efficient Meerwein–Ponndorf–Verley Reduction

**DOI:** 10.1021/acsami.3c16732

**Published:** 2024-02-26

**Authors:** Gábor Varga, Thanh-Truc Nguyen, Jing Wang, Dihua Tian, Run Zhang, Li Li, Zhi Ping Xu

**Affiliations:** † Australian Institute for Bioengineering and Nanotechnology, The University of Queensland, St. Lucia, Queensland 4072, Australia; ‡ Interdisciplinary Excellence Centre, Department of Applied and Environmental Chemistry, University of Szeged, Rerrich Béla tér 1, Szeged H-6720, Hungary; § Key Laboratory of OptoElectronic Science and Technology for Medicine of Ministry of Education, Fujian Provincial Key Laboratory of Photonics Technology, Fujian Normal University, Fuzhou 350117, China

**Keywords:** transfer hydrogenation, solid frustrated Lewis pairs catalysts, layered double hydroxides, isomorphic substitution, Ce(III)/Ce(IV) insertion, cyclohexanone reduction

## Abstract

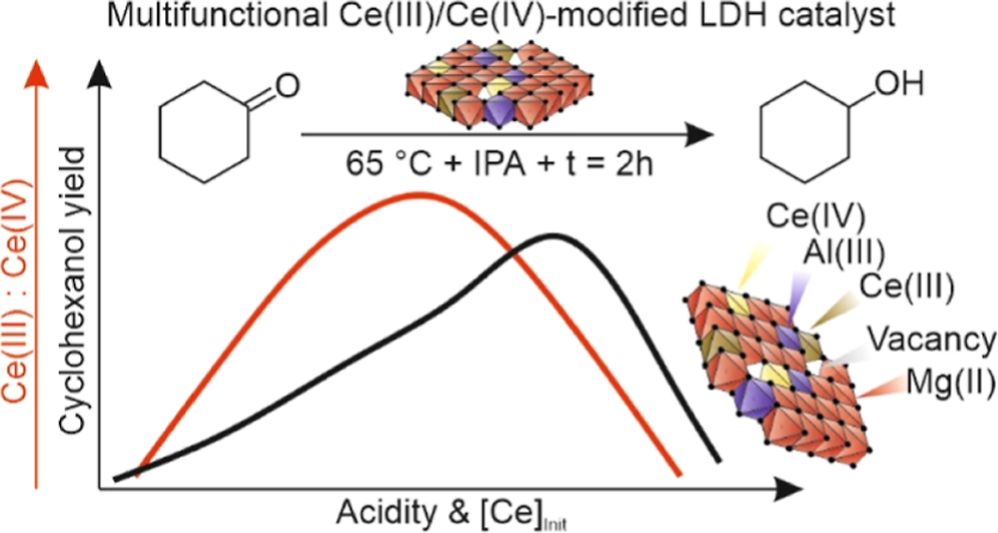

The development of highly active acid–base catalysts for transfer hydrogenations of biomass derived carbonyl compounds is a pressing challenge. Solid frustrated Lewis pairs (FLP) catalysis is possibly a solution, but the development of this concept is still at a very early stage. Herein, stable, phase-pure, crystalline hydrotalcite-like compounds were synthesized by incorporating cerium cations into layered double hydroxide (MgAlCe-LDH). Besides the insertion of well-isolated cerium centers surrounded by hydroxyl groups, the formation of hydroxyl vacancies near the aluminum centers, which were formed by the insertion of cerium centers into the layered double hydroxides (LDH) lattice, was also identified. Depending on the initial cerium concentration, LDHs with different Ce(III)/Ce(IV) ratios were produced, which had Lewis acidic and basic characters, respectively. However, the acid–base character of these LDHs was related to the actual Ce(III)/Ce(IV) molar ratios, resulting in significant differences in their catalytic performance. The as-prepared structures enabled varying degrees of transfer hydrogenation (Meerwein–Ponndorf–Verley MPV reduction) of biomass-derived carbonyl compounds to the corresponding alcohols without the collapse of the original lamellar structure of the LDH. The catalytic markers through the test reactions were changed as a function of the amount of Ce(III) centers, indicating the active role of Ce(III)–OH units. However, the cooperative interplay between the active sites of Ce(III)-containing specimens and the hydroxyl vacancies was necessary to maximize catalytic efficiency, pointing out that Ce-containing LDH is a potentially commercial solid FLP catalysts. Furthermore, the crucial role of the surface hydroxyl groups in the MPV reactions and the negative impact of the interlamellar water molecules on the catalytic activity of MgAlCe-LDH were demonstrated. These solid FLP-like catalysts exhibited excellent catalytic performance (cyclohexanol yield of 45%; furfuryl alcohol yield of 51%), which is competitive to the benchmark Sn- and Zr-containing zeolite catalysts, under mild reaction conditions, especially at low temperature (*T* = 65 °C).

## Introduction

Both the reduction of carbonyl compounds^1^ and the oxidation (dehydrogenation) of alcohols,^2^ especially secondary alcohols, are of particular importance from an industrial point of view. Although there are well-established processes for carrying out these basic conversions, these conversions suffer greatly from being time-consuming, wasteful, and requiring isolation/purification steps to remove undesired products and byproducts.^1,2^ These aspects are far from the requirements of green chemistry,^3^ and the emerging demand to optimize the redox economy of the synthesis processes, i.e., to minimize the number of oxidation state changes throughout the process,^4^ has led to the golden age of catalytic transfer hydrogenations (CTHs).^5,6^ Recently, this trend has been further strengthened as CTH reactions play a key role in many catalytic reaction cascades for the conversion of biomass-derived molecules into valuable products, which can greatly contribute to achieving the main goals of the circular economy.^7−13^

CTHs enable easy-to-use, affordable carbonyl reductions as well as alcohol oxidations even if in one-pot reactions.^5,7^ Moreover, they offer many advantages, from their well-controlled selectivity, including stereoselectivity (asymmetric transfer hydrogenations), and waste-minimized/time-spare features to the fact that they can be easily performed with heterogeneous catalytic processes, which increases their competitiveness.^5,7^ From a mechanistic point of view, two different types of intermolecular CTHs has been distinguished, i.e., the metal hydride route^14^ and the direct hydrogen transfer route.^15^ When the direct hydrogen transfer takes place between the α-H of the alcohol, which serves as the hydrogen source, and the carbonyl carbon via the formation of a six-membered-ring intermediate, this type of transfer hydrogenations is generally referred to as the Meerwein–Ponndorf–Verley (MPV) reduction.^7,15^ In heterogeneous catalysis, processes that follows this latter mechanism are proved to be even more useful than other transfer hydrogenation strategies.^5,7,15^ MPV catalysts typically have bifunctional features, particularly an acid–base character.^7,16^ In the homogeneous phase, Lewis alkoxides with electron-deficient metal centers [i.e., Al(*i*-Pro)_3_ or Ti(*i*-Pro)_3_] play the role of the promoter.^16^ It is noteworthy that both Lewis acid and base sites promote MPV, their coexistence being sufficient.^5,7,16^

Despite all the desirable progresses made, homogeneous catalysts have possessed considerable disadvantages due to the difficulties of ligand exchange, making the use of ligand excess sufficient, the separation of the catalysts from the products, and their sensitivity to water.^17^ There is therefore a notable need to find viable heterogeneous promoters that could overcome these difficulties.^18,19^ The development of highly efficient Sn- and Zr-substituted zeolites with isolated Lewis acid centers has given a decisive impetus to CTH technology, as they are still among the most attractive catalysts for MPV reactions today. Studying their catalytic performances, it becomes clear that the partially hydrolyzed oxide surfaces,^20,21^ which are consequently enriched with hydroxyl sites, are considered as active centers in these systems. Well-isolated active sites outperform those that are interconnected, and both the accessibility (*e.g*., open Sn sites) of the active sites^20,22^ and the hydrophobicity of the active surface have a remarkable influence on their catalytic performance.^20^ The presence of the hydroxyl groups is particularly important since the formation of reactive alkoxides can only occur with the aid of these groups.^20−22^ This phenomenon can be readily related to the fact that strong Lewis acid and base sites cannot coexist on the same catalyst when using these zeolitic-based catalysts.^18,23^

The lack of this ability of MPV catalysts has motivated studies to develop new strategies based on the concept of heterogeneous Frustrated Lewis Pairs (FLP) or FLP-like catalysis.^24,25^ In contrast to the popularity of homogeneous FLP catalysis,^24^ few studies have focused on developing its heterogeneous counterpart,^24−26^ and this concept is strongly limited to the application of CeO_2_-derived catalysts.^25−28^ Remarkably, the first results obtained in the context of solid FLP-catalyzed CTH have demonstrated that an active, selective, and robust CeO_2_-based catalyst enabled the conversion of various carbonyl compounds, albeit under harsh reaction conditions (>alcohol excess of 100-fold, *T* = 160 °C, high autogenous pressure, *t* = 24 h).^27,28^ To further improve the catalytic performance of CeO_2_, there are some significant challenges that are difficult to overcome. Particularly, both the generation of the defect sites and hydroxyl groups, the correct spatial distances between the active sites, and the stability of the active surface under the reaction conditions are not yet properly ensured, especially in a reproducible manner.^25,28^ Furthermore, by increasing the Ce(III)/Ce(IV)-ratio of a ceria surface to facilitate MPV reactions, the surface charge (ξ-potential) of the solid decreases and probably makes the adsorption of fully or partially negatively charged alcohols (alcoholates) more difficult, similar to the effect described for the nanozymatic applications of ceria.^29^

Given the results based on Zr/Sn-modified zeolite catalysts, it is hypothesized that isolated, well-established Ce(III)–O(H) centers inserted into a properly chosen host structure promote MPV reactions and outperform ceria. For this purpose, the use of nonactivated hydrotalcite [Mg_3_Al layered double hydroxide [named as layered double hydroxides (LDH))] as a host structure would be a good option. LDH is built on brucite (Mg(OH)_2_)-like hydroxyl layers in which part of the Mg(II) centers are isomorphic substituted by trivalent cations [*e.g*., Al(III), Ga(III), Fe(III), and Ce(III)].^30,31^ Due to this substitution, the hydroxyl layers have positive charges, which are balanced by the interlamellar anions (*e.g*., OH^–^, CO_3_^2–^*etc.*).^32−34^ With the cosubstitution of cerium cations in this structure [in addition to aluminum(III) centers], the stabilization of the Ce(III) oxidation state is expected to be similar to other valence-variable cations,^35,36^ and the adequate isolation of the active sites associated with the required hydroxyl groups is possible based on the even cation distribution model. Furthermore, LDH has constant positive surface charge (∼+30 to 40 mV), which is highly independent from the guest cations cosubstituted in a small extent.^32,37^ It is noteworthy that many studies have attempted to synthesize Ce-containing LDH.^38,39^ However, to the best of our knowledge, it has not been possible to produce a phase-pure LDH structure when more than 1% cerium compared to the aluminum centers is used. This must be related to the relatively high ionic radius of cerium compared to Mg/Al cations, which makes substitution less favorable.^40^ Fortunately in our group, there is a well-established procedure to insert high ionic radius cations [*e.g*. Gd(III)]^41^ into the LDH structure, also in a procedure that protects lower oxidation states of the cations from oxidation.^35,36^

In this paper, a useful optimized strategy to produce Ce-containing, phase-pure LDH is shown. We present that selective MPV reductions can be enabled under ambient conditions by well-isolated Ce(III) active specimens inserted into LDH structures, and these active centers overperform the catalytic ability of inserted Ce(IV) specimens. The crucial role of both the surface hydroxyl groups and the structural distortion/defect sites in the LDH structure generated by the isomorphic substitution are elucidated. A clear relationship between the acid/base property of the modified LDH structures and their catalytic performances is also depicted.

## Experimental Part

### Materials

All the chemicals were of reagent grade and were purchased from Merck or Sigma-Aldrich; they were used without further purification.

### Preparation of Hydrotalcites

Ce-containing magnesium–aluminum LDH (hydrotalcites, LDHs) were synthesized by a simple coprecipitation method (named in the text as one-step procedure). MgCl_2_ × 6H_2_O (3 mmol), (1 – *x*) mmol AlCl_3_ × 6H_2_O, and *x* mmol CeCl_3_ × 7H_2_O (*x* = 0.01–0.15 mmol) were first dissolved in 15 mL water. This mother liquor was then quickly added to 20 mL of freshly prepared NaOH solution of 0.40 M under a N_2_ atmosphere. After vigorous stirring for 45 min, the slurry obtained was centrifuged (4750 rpm for 5 min), washed twice with water and dispersed again (in 20 mL of water), and centrifuged (4750 rpm at 4 °C for 15 min after the first step and for 30 min after the second step). The prepared gel-like product was then thoroughly redispersed in 30 mL of water and stored at room temperature for 3 days. Thereafter, the solidified final product was separated by centrifugation (4750 rpm at 4 °C for 15 min) and then dried at 75 °C in vacuo for 16 h. The LDHs obtained are labeled as MgAlCe_*x*_, where *x* is the initial Ce(III)/Al(III) molar ratio: MgAlCe_0.01_/MgAlCe_0.15_. Pure hydrotalcite (Mg_3_Al-LDH; denoted as MgAl) was prepared in the same method in the absence of cerium salt, using 10 mL of mother liquor and 20 mL of 0.4 M NaOH solution.

The one-step method described above was used to synthesize phase-pure products, but the method was severely limited in terms of the number of cerium centers. To overcome this limitation, a second two-step preparation method was introduced. In this method, exactly the same reaction steps as described above were repeated, until the final suspension was obtained. In this case, before the final step (separation/drying), the entire batch of cerium-containing slurry suspended in water (30 mL) was placed in a Teflon-lined stainless-steel autoclave with a capacity of 50 mL and then heat-treated at 110 °C for 16 h, followed by the final separation procedure. This method allowed the incorporation of cerium in a slightly extended concentration range [up to a cerium content of 15% compared to that of the Al(III) centers].

### Characterization Methods

Powder X-ray diffraction (XRD) patterns of the solids were recorded on a Bruker D8 Advance powder XRD instrument by applying Cu Kα radiation (λ = 0.15418 nm) and 40 kV accelerating voltage at 40 mA in the range of 2θ = 5–80°. Dynamic light scattering (DLS) was used to measure the hydrodynamic size of the dispersed particles. The measurements were carried out with the same Nanosizer (Malvern) device as above at a 175° scattering angle in disposable plastic cuvettes.

The instrument for taking the Fourier-transform infrared (FT-IR) spectra was a Nicolet 5700 FT-IR spectrometer (Thermo Electron Corporation) with a 2 cm^–1^ resolution in attenuated total reflection mode (ATR-FT-IR). Raman spectra were recorded with a portable IM-52 Raman Microscope (Snowy Range Instruments). The 785 nm laser wavelength with a laser power of 70 mW was used for excitation of Raman scattering. The first coordination sphere and oxidation state of the transition metal ions was established by using an X-ray photoelectron (XP) spectroscopic mapping. XP spectra (XPS) were recorded with a SPECS instrument equipped with a Kratos Axis Supra Plus XPS, under a main-chamber pressure in the 10^–9^ to 10^–10^ mbar range.

Nitrogen sorption isotherms of samples were obtained using a Quantachrome Autosorb-1 analyzer at 77 K. The [Brunauer–Emmett–Teller (BET)] specific surface areas were calculated using adsorption data at a relative pressure range of *p*/*p*_0_ = 0.05–0.25. The morphologies of the samples prepared were studied by scanning electron microscopy (SEM). The SEM images were recorded on an FEI Quanta 650 FEG at an acceleration voltage of 20.0 kV. The thermal behavior of the as-prepared layered composites was studied on a thermogravimetric analysis/differential scanning calorimetry 1 STARe System (Mettler-Toledo Ltd., AU).

The amount of Ce, Mg, and Al components in the nanoparticles was determined by inductively coupled plasma-atomic emission spectrometry (ICP-AES) using a Varian Vista Pro instrument. Ce(III) content and Ce(III)/Ce(IV) actual molar ratios were determined by fluorescence spectroscopy (FLS) measurements using a SHIMADZU RF-5301PC spectrometer with excitation and emission slits of 5 nm. The measurements were carried out at room temperature using λ_ex_ = 255 nm and λ_em_ = 355 nm.

### Cyclohexanone Transfer Hydrogenation to Cyclohexanol in 2-PrOH

The MPV reactions of cyclohexanone, which were used as test reactions to describe the catalytic performance of the as-prepared solids, were carried out in a batch reactor at a constant reaction temperature of 65 °C under a N_2_ atmosphere. A solution of cyclohexanone (*c* = 0.5 M) and the appropriate amount of 2-PrOH (2–4 mL) was stirred under the above-mentioned reaction conditions in the presence of a chosen hydrotalcite derivative (50–200 mg) for an appropriate reaction time (1–360 min). When the reaction was completed, the obtained slurry was cooled down to room temperature and then centrifuged at 10,000 rpm for 10 min to remove the catalyst. The obtained mixture was then evaporated under reduced pressure and redissolved in 2-PrOH. Cyclohexanone conversions were determined by ultraviolet–visible (UV–vis) spectrophotometry in 2-PrOH on using the absorption band maximum of the cyclohexanone in the UV region (λ_max_ = 282 nm). The UV–vis absorbances were detected on a SHIMADZU UV-2450 spectrophotometer. For determining the cyclohexanol yields, ^1^H NMR measurements were introduced (Figure S1B,C). ^1^H NMR spectra were recorded at room temperature on a Bruker AV-500 in DMSO-*d*_6_.

## Results and Discussion

### Proof of Concept

In order to confirm the underlying hypothesis, initial transfer hydrogenation of cyclohexanone as the model reaction (Figure S1 and Scheme S1) was investigated in the presence of the as-prepared, phase-pure MgAlCe_0.05_-LDH, which was chosen arbitrarily at this point. To put its catalytic performance into the right context, the obtained data were compared with other benchmark catalysts and building blocks of the composites (Table 1).

**Table 1:**
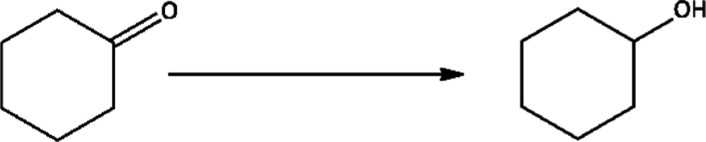
MPV Reduction of Cyclohexanone Catalyzed by As-prepared Ce-Containing LDH and Benchmark Catalysts^a^

	catalyst	cyclohexanone conversion (mol %)^b^	selectivity (mol %)^c^	cyclohexanol yield (mol %)^d^
**1**		<1		
***2***	*MgAlCe*_*0.05*_*–LDH*^e^	*10 ± 1*	100	*10 ± 0.9*
**3**	MgAl–LDH^e^	<1		
**4**	Cal. MgAl–LDH^f^	13 ± 0.8	100	13 ± 1.0
**5**	Reh. MgAl–LDH^g^	11 ± 0.7	100	11 ± 0.5
**6**	CeO_2_	4 ± 1	83	3 ± 0.4
**7**	NP CeO_2_^h^	7 ± 1	98	7 ± 0.7
**8**	ZrO_2_	35 ± 2	99	35 ± 0.9
**9**	Hyd. ZrO_2_^i^	45 ± 1	99	45 ± 1.4
**10**	MgO	6 ± 0.5	100	6 ± 0.4

a (Reaction conditions: *c*(cyclohexanone) = 0.5 M; *V*(2-propanol) = 4 mL; *m*(catalyst) = 100 mg; *T* = 65 °C; *t* = 5 h under N_2_ atmosphere.).

b Determined by UV–vis spectroscopy (*n* → π*; λ = 270 nm).

c Calculated: [(yield/conversion) × 100%].

d Determined by NMR spectroscopy.

e Modified coprecipitation (detailed recipe can be seen in Supporting Information).

f Calcined (*T* = 500 °C).

g Calcined-rehydrated.

h CeO_2_ nanoparticles.

i Hydrated ZrO_2_.

As determined, no reaction occurred in the absence of any catalysts under the set reaction conditions. It is noteworthy that more or less standard conditions were chosen for this comparison but the reaction temperature applied (*T* = 65 °C) was different from the standard one (*T* ≥ 82 °C).^42^ Apart from the green chemistry point of view, the LDH host probably has a capability of promoting the self-condensation of cyclohexanone, which is favored at higher temperatures.^43^

As can be seen in rows 3–7, nonmodified, nonactivated LDH and CeO_2_ did not have any notable activity to catalyze this reaction.^44^ As expected, at this reaction temperature, CeO_2_-based catalysts were not highly active, while the inactivity of LDH can be readily associated with the lack of the appropriate amount of accessible Lewis acid centers on their surface.^27,28^ After performing the activation procedure of LDH, the obtained mixed oxides exhibited moderate activity with exclusive selectivity exceeding the performance of a pure MgO catalyst.^45^ The tested ZrO_2_-type solids appeared to be highly active and selective (35–45 mol % cyclohexanol yield),^46^ with the performance depending on the pretreatment of the catalyst, which is in good agreement with the literature data.^22^ Upon using cerium-modified LDH as a promoter (Row 2), a moderate cyclohexanol yield of ∼10% with remarkable selectivity was achieved. This performance surpassed the capability of both nonactivated LDH and CeO_2_ as well as MgO and was close to the efficiency of the LDH-derived mixed oxides. As confirmed by the test reactions, the presented concept of interplay between isolated cerium centers and nonactivated LDH or activated LDH as the host is a viable alternative to useful MPV catalysts.

### Characteristics of As-prepared Ce-LDHs

**Figure 1:**
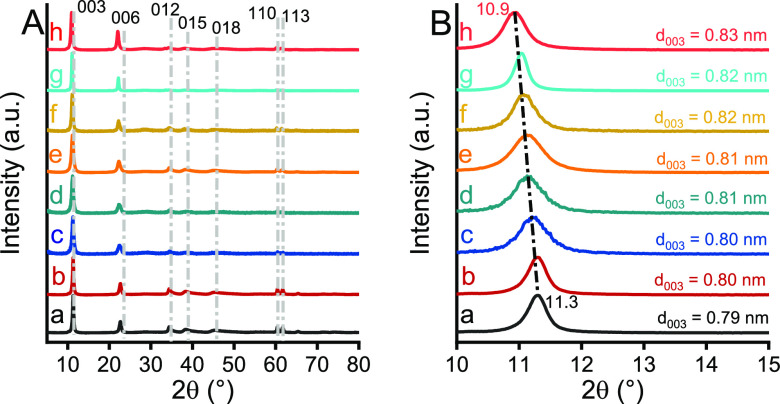
XRD patterns of the as-prepared (a) MgAl, (b) MgAlCe_0.01_, (c) MgAlCe_0.025_, (d) MgAlCe_0.05_, (e) MgAlCe_0.075_, (f) MgAlCe_0.1_, (g) MgAlCe_0.125_, and (h) MgAlCe_0.15_ in the 2θ range of (A) 5–80° and (B) 10–15° (MgAlCe_0.125_ and MgAlCe_0.15_ were synthesized via a two-step procedure, see Supporting Information).

By using the well-established protocol developed previously in our group (one-step procedure), cosubstituted—in this case cerium-modified LDHs—phase-pure, highly crystalline solid samples were prepared [marked as MgAlCe_*x*_, *x* = *n*(Ce)/(*n*(Al) + *n*(Ce)]. The characteristic XRD patterns of these solids closely resemble that of phase-pure LDHs with the rhombohedral structure, which can be assigned into the layered double hydroxide of the 3R_1_ polytype (Figure 1A; JCPDS card no. 89-0460). In contrast, as compared to the characteristic reflections of nonmodified LDH (labeled as MgAl) synthesized in the same method, a significant shift into lower 2θ region of the XRD patterns of the solid products was observed, indicating the increase in the interlayer distance (Figure 1B). Thus, this change can be identified as an increase in the hydroxide layer thickness as a result of the larger cerium incorporation.^47^ Moreover, the magnitude of this shift appeared to be proportional to the cerium content in the composites, confirming our assumption.

It is worth noting that cerium cations cannot be incorporated into MgAl in arbitrary amounts. There is a maximum initial cerium ratio of 10% to the total number of M(III) cations. Above this initial concentration, CeO_2_ (JCPDS no. 34-0394) was formed as the second phase in all cases (Figure S2). This Ce amount range was extended to a maximum of 15% when a coprecipitation was performed and followed by hydrothermal treatment (two-step procedure). The use of values above this extended range is practically accompanied by the presence of ceria as a byproduct without any exception.

To confirm the cerium insertion and determine the actual molar ratio of Ce-to-(Al + Ce), the ICP-AES measurement was applied. As seen in Table 2, each modified sample contained cerium in a different amount and the actual ratios were indeed close to the initial ones (incorporation rate = 95–98%) when the one-step procedure was used. However, a slightly larger difference was observed for the maximum initial concentration of cerium (90%). Those rates were significantly lower (75–81%) for the products prepared using the two-step procedure. It should also be mentioned that the actual Mg/M(III) ratio was almost identical to the theoretical one (3:1) in all cases. Furthermore, the TG/DTG analysis of the products showed that there were no remarkable changes in the actual composition of the LDHs compared to one another and to their nonmodified counterpart (Figure S3). Taking into account the results of the analytical methods presented, the most probable composition of the LDHs was calculated, as listed in Table 2. In addition, BET measurements confirmed that the specific surface area of the original MgAl structure did not vary significantly as a result of cerium insertion (Table 2, Figure S5).

**Table 2:** Actual Ce/Al and Mg/M(III) Molar Ratios, Interlamellar Water Content, and Calculated Compositions of the As-prepared LDHs

LDH composite^a^	Ce/(Al + Ce) ratio (%)^b^	Mg/M(III) ratio^c^	specific surface area (m^2^/g)^d^	interlamellar water ratio (m/m %)^c^	incorporation rate (%)^e^	composition^f^
MgAl	—	2.98	81	11	—	Mg_2.98_Al(OH)_7.96_(CO_3_)_0.5_ × 2.1 H_2_O
MgAlCe_0.01_	0.95	2.99	60	11	95	Mg_2.99_Al_0.9905_Ce_0.0095_(OH)_7.98_(CO_3_)_0.5_ × 1.9 H_2_O
MgAlCe_0.025_	2.43	2.97	85	11	97	Mg_2.97_Al_0.9757_Ce_0.0243_(OH)_7.94_(CO_3_)_0.5_ × 2.5 H_2_O
MgAlCe_0.05_	4.82	2.98	73	11	96	Mg_2.98_Al_0.9518_Ce_0.0482_(OH)_7.96_(CO_3_)_0.5_ × 2.5 H_2_O
MgAlCe_0.075_	7.13	2.97	71	11	95	Mg_2.97_Al_0.9287_Ce_0.0713_(OH)_7.94_(CO_3_)_0.5_ × 2.4 H_2_O
MgAlCe_0.1_	9.03	2.96	80	11	90	Mg_2.96_Al_0.9097_Ce_0.0903_(OH)_7.92_(CO_3_)_0.5_ × 2.4 H_2_O
MgAlCe_0.125_	10.10	2.95	84	11	81	Mg_2.95_Al_0.8990_Ce_0.1010_(OH)_7.90_(CO_3_)_0.5_ × 2.4 H_2_O
MgAlCe_0.15_	11.25	2.95	69	11	75	Mg_2.95_Al_0.8875_Ce_0.1125_(OH)_7.90_(CO_3_)_0.5_ × 2.3 H_2_O

a Ce_*x*_ (*X* = *n*(Ce)/*n*(Al) × 100%).

b Determined by ICP-AES.

c Determined by TG/DTG.

d Determined by BET measurements.

e Success rate = (*n*(Ce)_initial_/*n*(Ce)_actual_) × 100%.

f Calculation based on ICP-AES and TG/DTG results.

**Figure 2:**
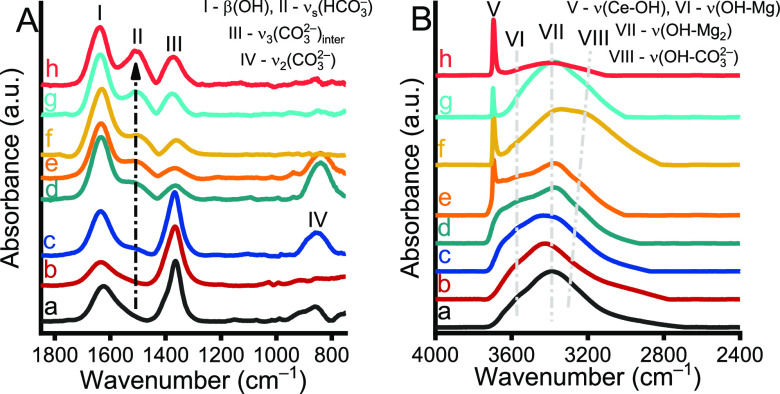
ATR-FT-IR spectra of (a) MgAl, (b) MgAlCe_0.01_, (c) MgAlCe_0.025_, (d) MgAlCe_0.05_, (e) MgAlCe_0.075_, (f) MgAlCe_0.1_, (g) MgAlCe_0.125_, and (h) MgAlCe_0.15_ in the wavenumber range of (A) 1850–800 and (B) 4000–2400 cm^–1^.

Moreover, the ATR-FT-IR spectra (Figure 2) provide evidence for the presence of the carbonate as charge compensating anions, represented by characteristic absorption bands at 1363 (ν_3_ CO_3_^2–^); peak III and 863 cm^–1^ (ν_2_ CO_3_^2–^), peak IV.^33^ However, considering our previous findings,^33^ it could be excluded that a significant amount of surface adsorbed carbonate would be enriched, which has a readily identifiable characteristic band around 1405–1410 cm^–1^ (Figure S4). This fact is of great importance because nonactivated LDHs often show lower catalytic activity due to the presence of counterions on the surface that mask the active (hydroxyl) sites.^48^ Besides, IR spectra include well-known, overlapped absorption band of water molecules and hydroxyl functions (peaks I, V–VIII).^33^ It can be seen that in the high wavenumber region (Figure 2B), both the peak positions and the intensities for various O–H bonds changed significantly when increasing the cerium content, which underpins the fact of the isomorphous cosubstitution.^49^

**Scheme 1:**
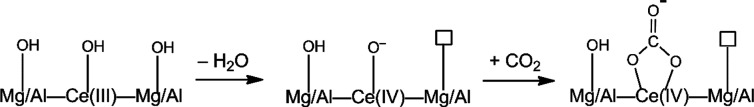
Plausible Mechanism of the Formation of Surface Defect Sites and Surface Adsorbed Bidentate Carbonate Species (Bicarbonate May Form via the Hydration of Bidentate Carbonate Specimen; Surface Defect Sites are Marked with Small Square)

More interestingly, a new vibration band around 1510 cm^–1^ came in the spectra of cerium-containing LDHs (Figure 2A; peak II), which is increasing in intensity with increasing cerium-content. It is difficult to identify this band beyond doubt, but probably it can be associated with the stretching mode vibrations of bicarbonate or bidentate carbonate specimens bound to the surface.^50^ Obviously, the occurrence of bicarbonate or bidentate carbonate specimens is not possible without the coexistence of Ce(IV)/defect centers.^50^ This can be illustrated using the analogies described for Ti^4+^ centers in TiO_2_ (Scheme 1).^51^ Nonetheless, it is difficult to state whether these Ce(IV)-containing specimens are inserted into the lattice or whether noncrystalline/smaller-sized CeO_2_ is formed to a lesser extent.

### Structural Features of the Ce-Insertion into an LDH Lattice

**Figure 3:**
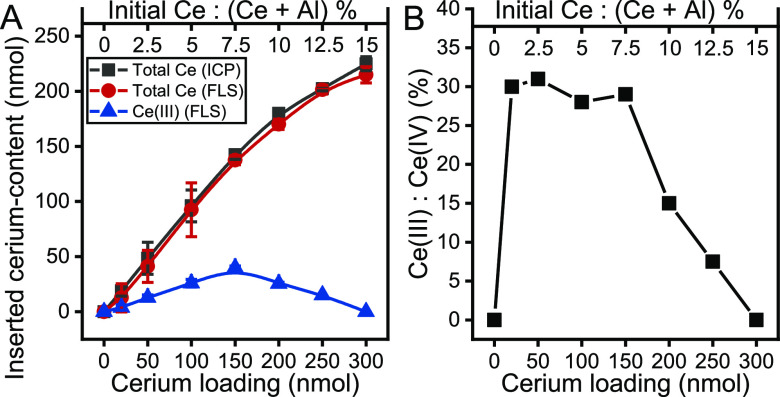
(A) Inserted total cerium-content and Ce(III)-content in the as-prepared LDHs (measured by ICP-AES and FLS) as a function of the initial cerium loading as well as (B) Ce(III)-to-Ce(IV) actual molar ratios in the as-prepared samples calculated from FLS results as a function of the initial cerium loading.

To prove the coexistence of Ce(III)/Ce(IV) centers and determine their actual molar ratio in the as-prepared LDHs, fluorescence spectroscopic (FLS) measurements were introduced in such a way that LDHs were dissolved in nonoxidative acid (Figure 3). The use of this method is based on the fact that Ce(III) specimens exhibit fluorescence in contrast to Ce(IV) species.^52^ As shown in Figure 3A, the determined total cerium concentrations, which was measured after the complete reduction of Ce(IV) ions with sodium sulfite,^53^ appeared to be in good agreement with the data measured by ICP-AES. The growth of the inserted total cerium amount was monotonic as a function of the initial cerium concentration (Figure 3A), regardless of the synthesis method used.

Otherwise, the cerium(III) concentration was found to change as a reverse “V” curve in relation to the initial cerium concentration, which had a maximum value at *x* = 7.5%. Furthermore, no detectable cerium(III) content was observed when this ratio of nominal replacement reached 15%, but there were more Ce(IV) specimens than Ce(III) in the samples (Figure 3B). In analogy to the insertion of Sn(IV) cations into LDH hosts, it is theoretically possible that these Ce(IV) centers are incorporated into the LDH lattice by isomorphic (co)substitution.

**Figure 4:**
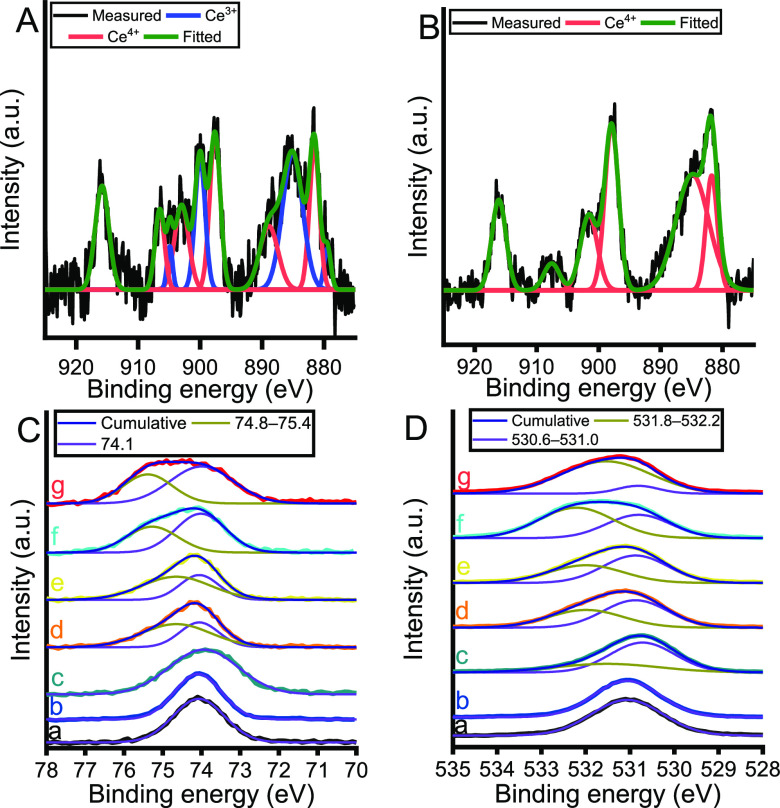
XP spectra of the Ce-containing LDHs. Ce_3d_ XP spectra of (A) MgAlCe_0.075_ and (B) MgAlCe_0.15_. (C) Al_2p_ and (D) O_1s_ XP spectra of (a) MgAl, (b) MgAlCe_0.025_, (c) MgAlCe_0.05_, (d) MgAlCe_0.075_, (e) MgAlCe_0.1_, (f) MgAlCe_0.125_, and (g) MgAlCe_0.15_.

XPS measurements were performed to determine the Ce(III)/Ce(IV) ratio on the active surfaces (Figure 4A,B; Figures S6 and S7; Table S4). The Ce_3d_ binding energy (BE) range of almost all Ce-containing LDH samples was fully described by including six well-known fitting components of Ce^4+^ specimens and four corresponding peaks of Ce^3+^ specimens surrounded by oxygen atoms in the fitted model (Figure 4A, S6, Table S4).^54^ It should be noted here that the MgAlCe_0.01_ sample was excluded from this XPS analysis, which had too low levels of cerium cations on the surface to allow the precise interpretation of the data. As for MgAlCe_0.125_ and MgAlCe_0.15_, the corresponding curves were fitted by considering only the peaks of the Ce^4+^ specimens (Figures 4B and S6). The change in the determined Ce(III)/Ce(IV) ratio (Figure S7) showed a good correlation with the trend ascertained by FLS.

Interestingly, the chemical environment of both the aluminum and oxygen atoms differs from that in pure LDH due to the isomorphic substitution. The Al_2p_ region of MgAl was readily described by only one parameter associated with the Al–O BE (74.1 eV) of the octahedrally coordinated, hydrated alumina lattice specimen in the MgAl-LDH (Figure 4C).^55^ On the contrary, at a nominal cerium loading higher than 5%, a second aluminum environment (BE of 74.8–76.4 eV) must be included in the model. This second environment strongly resembles that of tetrahedrally coordinated, partially hydrated alumina centers in MgAlCe-LDHs.^56^ This has indirectly proved the insertion of Ce(III)/Ce(IV) centers into the lattice and shown that the existence of the aforementioned defect sites (Scheme 1) is established. Parallel to this trend, a second chemical environment of oxygen specimens was also formed in the lattice (Figure 4D). It should be noted here that the O_1s_ XPS measurements further support the assumption that hydroxyl groups (BE ∼ 532 eV) can be found on the surface while the presence of the carbonate specimens can be excluded.^57^ This finding is further supported by the C_1s_ spectra of the LDHs (Figure S6).^57^ As for the O_1s_ region, there was no evidence for the existence of oxygen atoms in a chemical environment similar to that of CeO_2_.^54^ Accordingly, all of the cerium spectra obtained belongs to inserted cerium centers.

**Figure 5:**
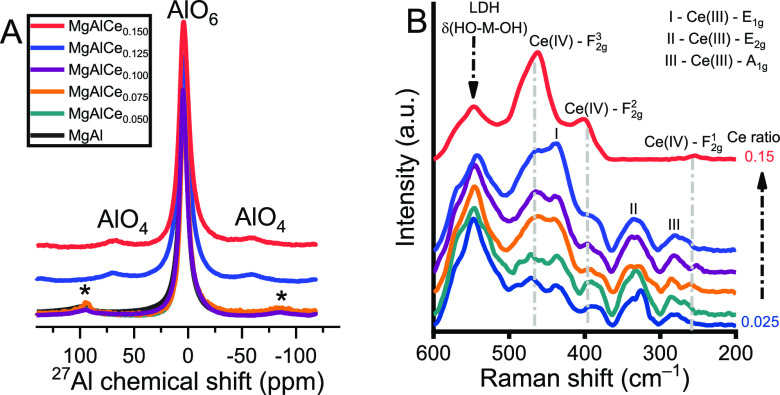
(A) ^27^Al-SS-NMR spectra and (B) Raman spectra of the as-prepared LDHs. (The color coding is the same in both graphs).

To determine whether tetrahedrally coordinated aluminum centers are truly formed in LDHs, as suggested in the XPS studies, ^27^Al-solid-state (SS)-NMR spectra were recorded (Figure 5A). A significant broadening of the basic peak associated with octahedrally coordinated lattice aluminum centers (∼4.38 ppm)^58^ was observed in the presence of cerium cations loaded to an extent of 1–7.5% compared to the aluminum centers in MgAl; otherwise there was no difference. Using a higher initial concentration of cerium, fingerprint-like chemical shifts around 70 ppm known to belong to the tetrahedrally coordinated aluminum centers appeared in the NMR spectra.^58^ These are likely formed by the incorporation of Ce(III)/Ce(IV) cations into the lattice (Scheme 1). However, these partially hydrated alumina centers should be considered as defect sites since they coexist in a double hydroxide layer surrounding with octahedrally coordinated, fully hydrated magnesium/aluminum centers. Note that the XPS method is a surface sensitive method, whereas the SS-NMR method is bulk sensitive. Accordingly, the observed trends of the two methods differ slightly due to quantitative differences in this case.

However, the existence of the specimens described above does not exclude the formation of other types of defect sites that could be related to the Ce(III)/Ce(IV) interaction. This can be readily verified by Raman spectroscopy (Figures 5B and S8). As reported by Schilling et al. and Liu et al.,^59,60^ Raman spectra allow us to distinguish the “real” oxygen vacancies near the Ce^4+^ centers (500 cm^–1^), the Ce^3+^ centers (or other vacancies) in the second coordination sphere of the Ce^4+^ centers (470–480 cm^–1^), and the CeO_2_-like environment (455 cm^–1^). In the Raman spectrum of pure LDH (Figure S8), there is only one broadened peak (545 cm^–1^) in the range (600–200 cm^–1^), which is due to a combination of bending mode vibrations of Al–OH and Mg–OH units [δ(OH–M–OH)].^61^ For comparison, both a physically mixed composite of MgAl-LDH and CeO_2_ and LDH-supported CeO_2_ prepared by wet impregnation were synthesized. In their Raman spectra (Figure S8), beside the peak of the LDH structure that remained, a new peak appeared at 460 cm^–1^, attributed to the F_2g_ vibrational mode of fluorite-type CeO_2_.^59,60^ All of the measured Raman spectra of the Ce-LDHs were fitted into seven different Raman bands (Figure 5B). As expected, the fingerprint-like band of the LDH lattice was readily found at a slightly shifted band position of 550 cm^–1^. Three other bands at 473, 390, and 260 cm^–1^ were associated with Ce(IV)-containing specimens, which are identified as F_2g_(3), F_2g_(2), and F_2g_(1) bands, respectively. Considering the red shift of the F_2g_(3) band, it is assumed that the Ce(IV) centers do not bind directly to defect sites but Ce(III) cations (or other defect sites) occupy part of the lattice positions in their second coordination sphere. Obviously, cerium(III) cations were incorporated into a chemical environment in hydroxide layers almost identical to that in Ce(OH)_3_/Ce(H_2_O)_*x*_^3–^, as reflected by three Raman peaks associated with the Raman bands A_1g_ (∼284 cm^–1^), E_2g_ (∼340 cm^–1^), and E_1g_ (433 cm^–1^) of the Ce(OH)_3_-like structure.^62^ Compared to the reported data, there was a clear blue shift in the E_1g_ band, which probably means that these centers are integral parts of the hydroxide layers. In the case of MgAlCe_0.15_, all three Raman bands of the Ce(III) specimens disappeared, consistent with the fluorescence data that the Ce(III)/Ce(IV) ratio was close to zero.

### Acid–Base Properties of the As-prepared Samples

Since MPV reactions are acid–base catalyzed reactions, it is critical to investigate the effects of cerium insertion in relation to the basicity and acidity of the solid samples.^63^ Thus, ATR-FTIR probing measurements were conducted using probe molecules, in particular pyridine^64^ to characterize the acidic centers and methanol^65^ to measure the basic centers.

**Figure 6:**
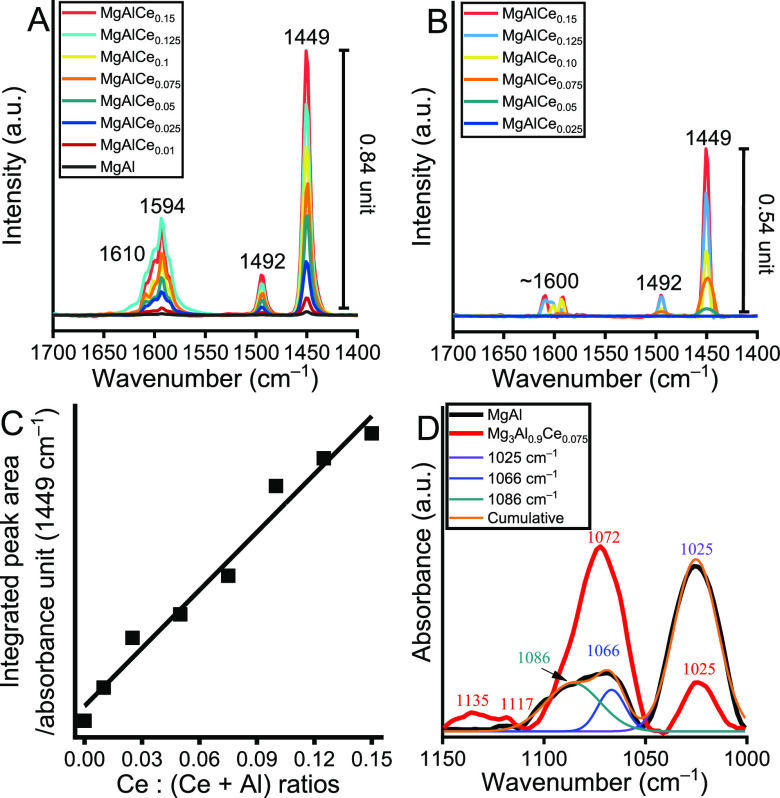
ATR-FT-IR spectra recorded after (A) adsorption of pyridine at room temperature on the samples and (B) adsorption of pyridine at room temperature on the samples followed by desorption at *T* = 125 °C. (C) Integrated peak area of the peaks at 1449 cm^–1^ as a function of initial Ce/Al ratios. (D) ATR-FT-IR spectra recorded after adsorption of methanol at room temperature on the samples. (All the presented spectra are difference spectra obtained after the subtraction of the corresponding spectra of the LDHs without adsorbed probe molecules.).

As shown in Figure 6A, four different characteristic absorption bands were observed in the spectra of pyridine adsorbed on MgAlCe samples at 1610, 1594, 1492, and 1449 cm^–1^ in the fingerprint region (1700–1400 cm^–1^). According to the literature report of pyridine adsorption,^64^ these bands are assigned to coordinatively bound pyridine (1449 and 1610 cm^–1^) adsorbed on Lewis acid centers and physisorbed pyridine (1594 cm^–1^). The absorption band at 1492 cm^–1^ is a combination band containing vibrational components of both coordinately bound pyridine and pyridinium ions produced by the reaction of pyridine and Brønsted acids. However, the presence of Brønsted acid sites could be ruled out since bands around 1638 and 1545 cm^–1^ were not observed at all. To confirm this assignment, the measurement was repeated after desorption at a higher temperature (*T* = 125 °C; Figure 6B). As expected, the peak intensity of the strongly bound pyridine samples (1610, 1492, and 1449 cm^–1^) decreased to varying degrees as a function of acid strength, while the peak associated with physisorbed pyridine decreased sharply in the intensity. It is noted that there were relatively large differences in the peak intensity of pure and Ce-modified LDHs, which were increased at higher outgassing temperatures. There were no peaks in the spectra of pure LDH and MgAlCe_0.01_ when a higher outgas temperature was used (not shown). As previously reported,^66^ pure, nonactivated LDHs have very weak Lewis acidity due to hydration of the basic Brønsted centers. This Lewis acidity is considerably enhanced by Ce insertion, which explains the increased catalytic activity during transfer hydrogenation. Plotting the integrated peak areas of the characteristic band of 1449 cm^–1^ as a function of the initial Ce:(Al + Ce) ratios in the LDHs revealed a linear trend (Figure 6C), indicating that both Ce(III) and Ce(IV) centers are accessible Lewis acid centers on the surface, and their active role will be reflected during the subsequent catalytic reactions.

Interestingly, cerium incorporation has also caused a slight change in basicity in LDHs (Figure 6D). Three characteristic adsorption bands were identified in the spectrum of the pure LDH at 1086, 1066, and 1025 cm^–1^ in the range 1150–1000 cm^–1^. These belong to the methoxy specimens of the bidentate linkage originating from the bond cleavage of the OH group of methanol (1086 cm^–1^; species II), undissociated methanol molecules adsorbed on Mg–OH units (1066 cm^–1^), and Al–OH units (1025 cm^–1^),^65^ respectively. Looking at the spectra of cerium-modified structures, a similar envelope of spectra with three components at positions of 1135–1117, 1072, and 1025 cm^–1^ was observed (Figures 6D and S9). The last two components show adsorption of undissociated methanol. More interestingly, the absorption bands above 1110 cm^–1^ can be attributed to dissociated methanol molecules, especially methoxy species with monodentate linkage (species I).^65^ Considering the generally accepted mechanism of MPV reactions, species I is of particular importance in the activation of hydrogen sources (alcohol molecules). Accordingly, apart from the significantly more Lewis acid centers in cerium-modified LDHs compared to those in pure LDH, this aspect is certainly important with regard to the increased catalytic activity.

### Optimization of Conditions for Ce-LDH Catalyzed MPV Reduction of Cyclohexanone

**Figure 7:**
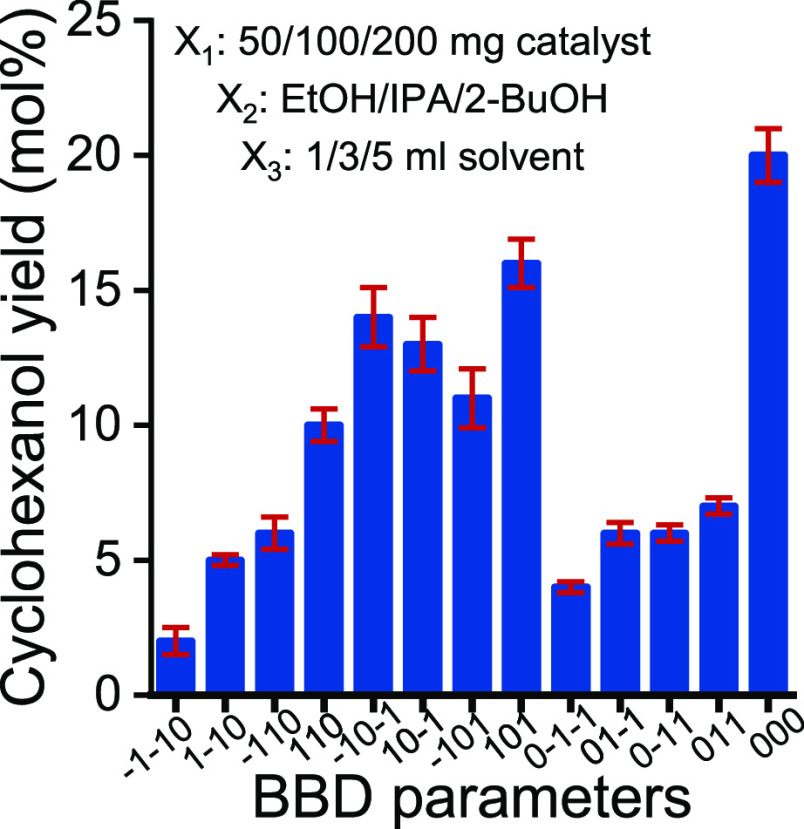
Experimental runs of the BBD. [Parameter web: *X*_1_: −1 = 50 mg catalyst (MgAlCe_0.05_); 0 = 100 mg catalyst; 1 = 200 mg catalyst; *X*_2_: −1 = EtOH (used as H-source and solvent); 0 = 2-propanol; 1 = 2-butanol; *X*_3_: −1 = 1 mL solvent; 0 = 3 mL; 1 = 5 mL].

By introducing a Box–Behnken design^67^ (BBD; Figure 7, Table S1) optimization procedure, the external reaction conditions [catalyst loading (50, 100, and 200 mg), hydrogen donor used (EtOH, 2-propanol and 2-butanol) and alcohol excess (1, 3, and 5 mL)] were optimized when the cyclohexanone concentration (*c* = 0.5 M), the reaction temperature (*T* = 65 °C), and the reaction time (*t* = 5 h) were fixed under an N_2_ atmosphere.

Clearly, the use of MgAlCe_0.05_ with a loading of 100 mg and an isopropanol volume of 3 mL maximized the cyclohexanol yield. Moreover, both ethanol and 2-butanol could serve as the hydrogen donor in this MPV reaction, but the use of 2-propanol significantly increased the yield. These BBD tests also confirmed that the catalyst efficiency started to decrease immediately under an oxidative atmosphere, indicating the necessity of a N_2_ atmosphere during the catalytic reaction, probably related to the oxidizable feature of Ce(III) centers (Figure S10) since characteristic reflections of CeO_2_ appeared in the XRD patterns of this LDH after the reactions (Figure S11). Under the optimized reaction conditions, a 20% cyclohexanol yield with 100% selectivity was achieved in the presence of MgAlCe_0.05_ as the catalyst. When this reaction was repeated in air, the cyclohexanol yield was reduced to 3%.

### Structure–Activity Relationship

**Figure 8:**
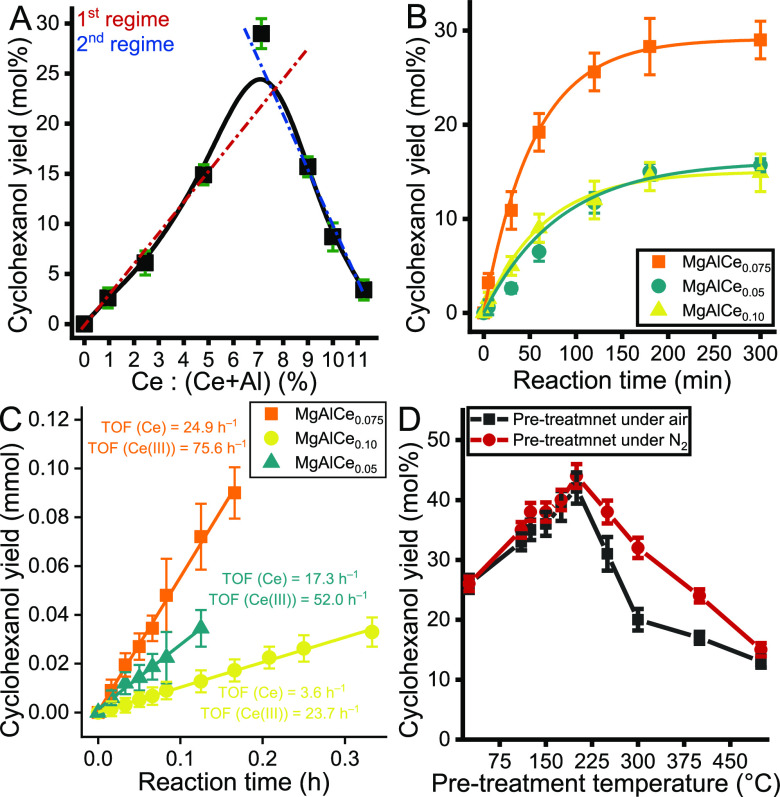
(A) Impact of the concentration of the inserted cerium centers on their own catalytic performance of the as-prepared LDHs toward transfer hydrogenation of cyclohexanone to cyclohexanol under the optimized reaction conditions [*c*(cyclohexanone) = 0.5 M; *V*(2-propanol) = 3 mL; *m*(catalyst) = 100 mg); *T* = 65 °C; under a N_2_ atmosphere] in 5 h. (B) Kinetic profile of the cyclohexanone-to-cyclohexanol reaction catalyzed by three different Ce-containing LDHs under the optimized reaction conditions. (C) Determination of TOF values of the chosen LDHs through cyclohexanone-to-cyclohexanol transformations under the optimized reaction conditions. (D) Impact of the (pre)heat treatment of the chosen catalyst (MgAlCe_0.075_) on its own catalytic performance in CTH of cyclohexanone-to-cyclohexanol reactions under the optimized reaction conditions in 3 h.

With the optimized reaction conditions in hand, we examined the effects of the structural features of the Ce-containing LDH structures on their catalytic performance. Repeatedly note that as-prepared phase-pure LDHs had very similar specific surface area (Table 2), crystallite size, and morphology (Figure S5 and Table S3). As shown in Figure 8A, the reaction marker (cyclohexanol yield) changed with increasing amounts of incorporated cerium in the LDH layers. The trend can be described with an asymmetric volcano-like curve, which has a maximum yield at a cerium ratio of 7.5% to the amount of total M(III) centers. In the first regime, the gradual increase of cerium centers inserted into LDH leads to improved activity. Accordingly, this regime was fitted into an almost perfectly linear trend line. Clearly in the second regime, the increase of the nominal Ce concentration over 7.5% decreased the yield. Following this observation, it seems a plausible explanation that the catalytic activity decreases in parallel with the decrease in the number of Ce(III) centers on the surface in this regime in a linear trend. However, this does not lead to a complete loss of catalytic activity, since the Ce(IV) centers also exhibit some catalytic activity for MPV reactions at a much lower level than Ce(III) centers.^68^ Same correlation between the activity of Ce(III) and Ce(IV) centers, in terms of transfer hydrogenation reactions, has already been observed in the homogeneous phase.^68^

Nevertheless, the catalytic performance of the optimized catalyst (MgAlCe_0.075_) does not fit into any of the two linear trends. It is assumed that in this Ce-LDH, predominantly Ce(III) centers should be considered as active sites, and aluminum-based defect sites already coexist on the surface of the catalyst to facilitate the catalytic performance. In this case, the interplay between Ce(III)/Ce(IV) and defect sites might be considered as FLP-like behavior. To support this assumption, the kinetic curves of the MPV reactions catalyzed by three different Ce-LDH catalysts were recorded (Figure 8B). The three catalysts were selected from three different regimes of the presented activity trend. All kinetic curves exhibit a well-formed sigmoidal growth trend, likely due to a pseudo second order kinetics. The first regime of these curves is linear enough to determine TOF values (Figure 8C). Considering all cerium centers as possibly active centers, the TOF values differ significantly. Repeating the calculation was repeated by considering only Ce(III) centers as active centers; almost the same differences were obtained. Different TOF values must be associated with the different number and activity of active centers.^18^ Thus, our assumption is partially proven because the accessibility of active centers, especially Ce(III) centers, may differ from each other in different samples depending on the Ce(III) number and the Ce(III)/Ce(IV) ratio. Nonetheless, this hypothesis can explain the fact that a cyclohexanol yield of 29% (25% within 2 h) was obtained via promoting the MPV reaction with MgAlCe_0.075_, which is very close to the efficiency of the benchmark ZrO_2_ catalyst.

To underpin our hypothesis that the existence of the FLP-like specimens on the surface, a combined O_2_-, NH_3_-, and CO_2_-T(emperature)P(rogrammed)D(esorption) study was initialized (Figure S12) focusing on the comparison of MgAl and MgAlCe_0.075_ samples.^28^ Both O_2_-TPD profiles consisted of two separately occurring desorption peaks in the relatively high temperature range of 380–650 °C (Figure S12A). These can be readily associated with the desorption of surface lattice oxygen (380–500 °C), while the bulk lattice oxygen desorbed above 500 °C. For MgAlCe_0.075_, a third desorption peak also appeared at 100–150 °C which is the desorption of surface chemisorbed oxygen specimens which are normally located at surface defect sites, particularly oxygen or hydroxyl vacancies. This indicates that hydroxyl vacancies exist on the surface of the MgAlCe_0.075_ catalyst and can act as Lewis acid sites. Furthermore, unsaturated Lewis acid sites near these defect sites tend to form FLP structures with Lewis base sites. The presence of Lewis base sites can be readily detected by CO_2_-TPD measurements (Figure S12B). As we can see, the CO_2_-TPD profile of MgAlCe_0.075_ is very similar to that of MgAl, as it contains only one desorption peak in the temperature range of 200–250 °C, which is related to the weak basic sites, probably −OH groups with a Lewis base character. However, its maximum is shifted to higher temperatures (200 → 220 °C) compared to that of pure hydrotalcite, indicating the presence of stronger basic sites. There are two desorption peaks in the NH_3_-TPD profile of MgAlCe_0.075_ at 180 and 310 °C (Figure S12C). Considering the lack of acidity of pure hydrotalcite (no desorption peak in its NH_3_-TPD profile),^69^ this is sufficient evidence that unsaturated Lewis acid centers can be found on the surface. If we add to this the proven coexistence of hydroxyl vacancies and slightly reinforced Lewis base centers, we are firmly convinced that FLP specimens form on the surface of the MgAlCe_0.075_ catalyst.

One of the main cornerstones of our hypothesis is that hydroxyl groups play an important role in catalytic performance. To demonstrate the correctness of this assumption, heat treatment of the catalyst at various temperatures in its initial state was performed (Figure 8D). As the observed changes were due to the conversion of Ce(III) → Ce(IV) on the surface, the pretreatment was carried out under a N_2_ atmosphere and air in each run for comparison. Based on the TG/XRD curves (Figures S3 and S13), the catalytic performance of the MgAlCe_0.075_ was expected to remain unchanged when the pretreatment temperature was changed until surface dehydroxylation occurred. Contrary to this assumption, pretreatment at 100 and 200 °C produced more active catalysts, with the cyclohexanol yield increasing to 33 and 42%, respectively (Figure 3). In this temperature range, two significant mass losses, i.e., the loss of adsorbed water (∼111 °C) and interlamellar water (∼200 to 270 °C), occurred. However, this dehydration did not cause the collapse of the long-range ordered layer structure of LDH. The observed increase in cyclohexanol yield is likely related to the exposure of active sites on the surface of the catalyst after the removal of these water molecules that associate with and block the OH active sites. At a pretreatment temperature above 250 °C, the structure of the modified LDHs gradually collapses, resulting from dihydroxylation of the active hydroxyl groups on the surface and leading to decreased yields. Moreover, a redistribution of the cation order probably also occurs at above 250 °C, as described in the literature on the thermal behavior of LDH.^70^ As a result, the new active surface is enriched in MgO specimens; thus, the surface properties do not meet the requirements of FLP catalysis, and the efficiency of the formed bifunctional mixed oxide catalyst is far from that of its counterpart in the initial state. Accordingly, the studied catalyst is probably considered a solid FLP-like catalyst.

### Heterogeneity, Recyclability, and Versatility of MgAlCe_0.075_ Catalyst

**Figure 9:**
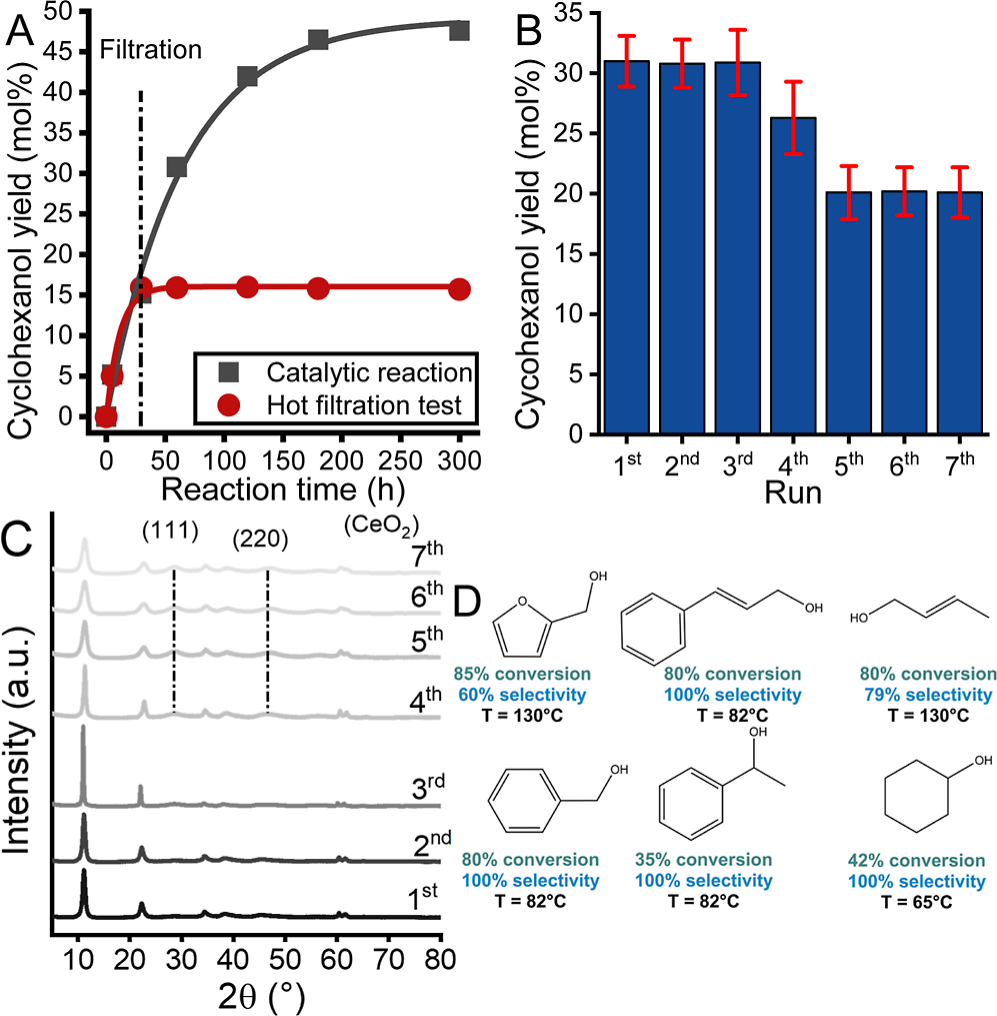
(A) Hot filtration and (B) recycling tests (reaction time: 1 h) of the heat treated (200 °C) MgAlCe_0.075_ solid in a cyclohexanone-to-cyclohexanol transfer hydrogenation. [Reaction parameters: *c*(cyclohexanone) = 0.5 M; *V*(2-propanol) = 3 mL; m (catalyst) = 100 mg; *t* = 3 h; *T* = 65 °C; under a N_2_ atmosphere]. (C) XRD patterns of the spent catalyst run-by-run. (D) Short scope of the catalytic system [main products are depicted in the order of furfuryl alcohol; cinnamyl alcohol, crotyl alcohol; benzyl alcohol; 1-phenylehtanol, cyclohexanol. Reaction time: 8 h except for cyclohexanol (2 h)].

The heterogeneity of MgAlCe_0.075_ pretreated at 200 °C was demonstrated by a hot filtration test (Figure 9A) performed under the optimized reaction conditions. The actual catalyst was removed from the reaction mixture after 30 min, and the reaction was then continued with the filtrate for another 90 min. As shown in Figure 9A, the catalytic reaction was stopped by filtering out the Ce-LDH catalyst from the system, indicating that no active specimens were leached into the reaction mixture and the catalysts used were a true heterogeneous catalyst.

The stability and recyclability of the catalyst were investigated by separating, washing, and reusing the spent catalyst after the successful MPV reduction under the optimized reaction conditions (except for the used reaction time: 1 h). The data presented in Figure 9B clearly show that the cyclohexanol yield remained constant in three consecutive runs and decreased by 5–10% overall in the fourth and fifth runs. No further decrease in activity was observed during further recycling. Remarkably, there were no changes in the unique selectivity of the catalyst during the recycling tests. By performing an ex-situ XRD study of the spent catalyst after different runs, a plausible explanation for the loss of activity in the corresponding runs was found (Figure 9C). After the fourth run, a characteristic reflection of CeO_2_ was observed in the corresponding diffractogram, which increased in intensity in the spent sample used in the fifth run. However, the intensity ratios between the LDH reflections and the CeO_2_ reflections became constant in the spent samples after the fifth run, in parallel with the restabilization of the cyclohexanol yields.

The remarkable activity of MgAlCe_0.075_ pretreated at 200 °C has encouraged us to extend our study to LDH-catalyzed MPV reductions of some other industrially important biomass-derived ketones and aldehydes (Figure 9D, S14, and Table S2). In these cases, a moderate-to-high conversion of carbonyl compounds was achieved within 8 h (2 h in the case of cyclohexanol) at different temperatures, depending on the quality of the reactants (Figure S14). In fact, the reaction temperature plays a crucial role due to the Brønsted/Lewis base property of the LDH framework. If a higher temperature was applied to promote the reaction, aldol condensation occurred as an undesirable side reaction, which reduced alcohol selectivity. Excitedly, the yield and selectivity seem to be competitive or even superior to the best available technologies to prepare the above listed biomass derived alcohols in these cases,^11^ demonstrating the versatility of the optimized catalyst, i.e., MgAlCe_0.075_, for MPV reductions.

### Mechanistic Insight of the MgAlCe_0.075_ Catalyzed MPV Reduction of Cyclohexanone

In order to propose an appropriate reaction mechanism, some control experiments were performed focusing on the catalytic ability of MgAlCe_0.075_ and its building blocks for oxidation/oxidative-dehydrogenative transformations of alcohols (benzyl alcohol, 1,2-hexanediol, and diphenylmethanol) (Scheme S2 and Table S5). This is the rate-determining step of the MPV mechanism. As can be seen, neither pure LDH nor its partially dehydrated counterpart can promote these transformations, highlighting their negligible role in these reactions. When cerium-containing catalysts were used, all of them catalyzed the conversion of alcohols except diol, as its oxidation is less favorable than that of primary or even secondary alcohols (Table S5, rows 1–3). In sharp contrast, when the tests were repeated under a N_2_ atmosphere, only hydrated nanoceria with surface defect sites and hydroxyl groups as well as FLP-containing CeO_2_ proved to be active catalysts (Table S5, rows 4–6). In addition, the same trend was observed in the presence of an acceptor molecule (aniline, Table S5, rows 7–9), using an N_2_ atmosphere. This means that in the case of a Ce-containing catalyst, the presence of an FLP-like structure^27^ and/or Ce(III) centers surrounded by hydroxyl functions^71^ are probably necessary in the dehydrogenation of alcohols under an inert atmosphere where Ce(IV) oxide-type catalysts are very inactive as is well-known based on the literature data.^72^ The best performance can be achieved when Ce(III) centers, hydroxyl groups with basic character, and defect sites are simultaneously present on the active surface (hydrated nanoceria). When the MgAlCe_0.075_ catalyst was included in these test reactions, it was found that its performance exceeded that of all Ce-containing (and also LDH-containing) catalysts and only the hydrated nanoceria could match its efficiency. Thus, similar active centers are found in both catalysts, but in MgAlCe_0.075_, the cerium centers are (possibly) much more isolated than those in hydrated nanoceria.

**Scheme 2:**
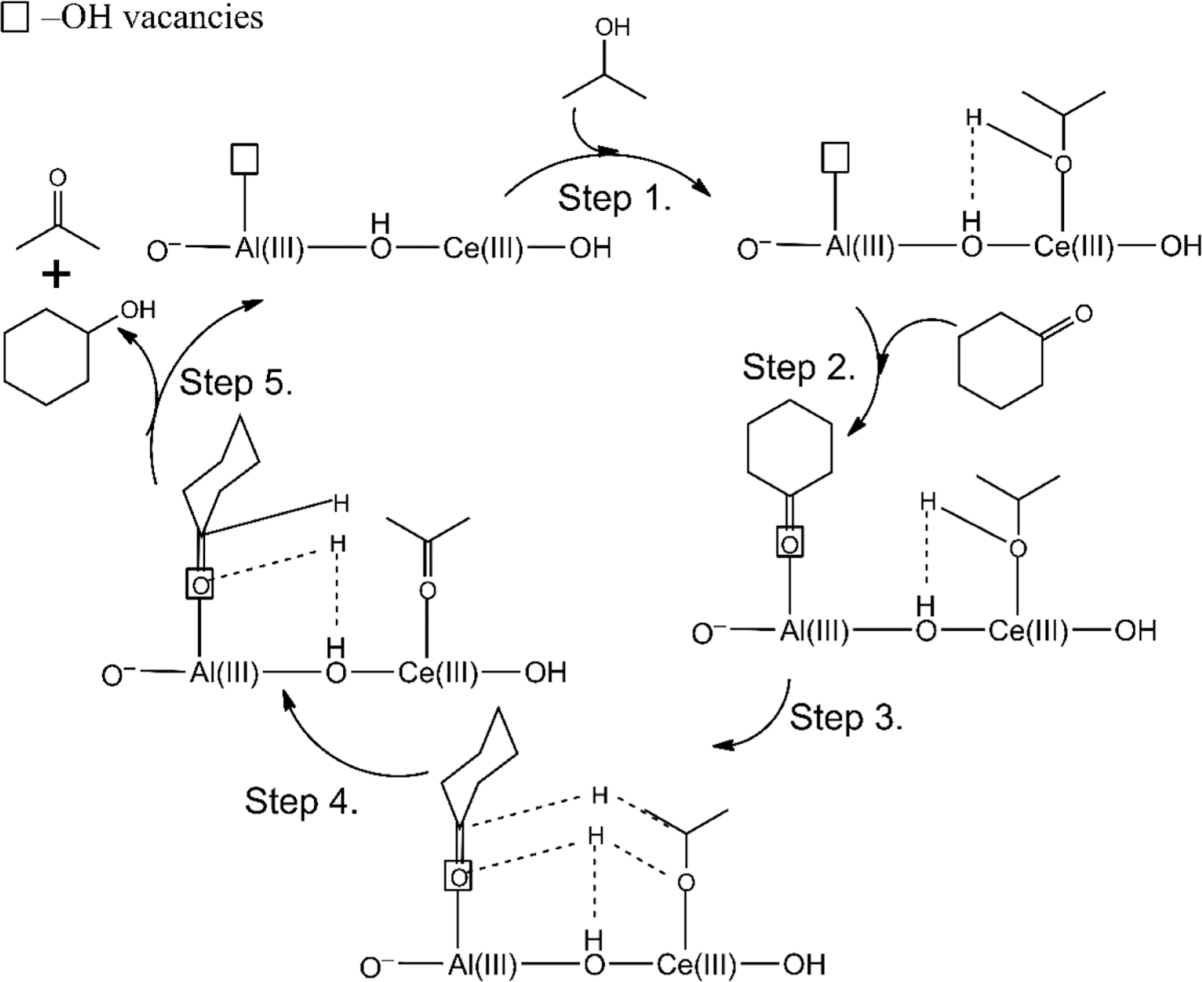
Proposed Mechanism of the Transfer Hydrogenation of Cyclohexanone to Cyclohexanol in 2-Propanol Catalyzed by the MgAlCe_0.075_ Catalyst

Based on all this information, a likely reaction mechanism for the Ce-LDH-catalyzed MPV reduction of cyclohexanone was proposed (Scheme 2), considering the results reported for high entropy oxides^28^ with FLP-like activity in combination with the classical acid–base mechanism.^7^ Accordingly, in the first step, the dissociative adsorption of the 2-propanol molecule onto a coordinatively unsaturated Ce(III) center takes place with the help of the framework −OH unit with Lewis basic character. This first step is supported by three facts. First, in most coordination compounds of cerium cations, the coordination number is between 7 and 9, so both Ce(III) and Ce(IV) in the LDH structure can be treated as coordinatively unsaturated centers,^73^ but are stabilized by the LDH lattice. Second, methanol adsorption studies have shown that the MgAl-LDH surface can adsorb alcohol molecules in a dissociative manner. Finally, through the control experiments, a catalyst having Ce(III)-containing centers in conjunction with the hydroxyl functions exhibited notable performance for promoting dehydrogenation of alcohols. The main role of Ce(III) centers in this step can be also supposed according to the comparative study of alcohol oxidations catalyzed by highly active Ce(III) complexes, which has also declared the reduced activity of Ce(IV) complexes.^74^ In the absence of Ce(III) centers, this step can also take place at Ce(IV) centers but with a low efficiency. In the second step, the electron-rich oxygen of the carbonyl group of cyclohexanone coordinates to an electron-deficient −OH vacancy with Lewis acidic features similar to a key step previously proposed for FLP-like surfaces. Then in the third step, similar to the classical MPV mechanism, the formation of a six-membered ring intermediate occurs, immediately followed by the transfer of both α-H and hydrogen of the OH group of the alcohol molecule to the carbonyl group in a concerted step. Finally in steps 4 and 5, desorption of the products (cyclohexanol, acetone) occurs, which also regenerates the active sites on the surface for the subsequent reactions.

## Conclusions

In conclusion, cerium-containing LDH can act as highly efficient heterogeneous catalysts to promote the MPV reduction of various ketones and aldehydes. Highly crystalline phase-pure Ce-containing LDHs (Mg_3_Al_1−*x*_Ce_*x*_-LDH) were prepared by using modified coprecipitation processes. In contrast to the previously reported synthesis, our method allowed the preparation of phase-pure Ce-LDHs, but with a severe limitation on the maximum amount of incorporated cerium centers (actual ratio of ∼11% of the total M(III) cations). By adopting a very broad characterization (XRD, TG/DTG, ICP-AES, FT-IR, BET, SEM, DLS, FLS, XPS, SS-NMR, and Raman), it was clearly demonstrated that Ce incorporation was successful, and both Ce(III) and Ce(IV) centers were formed in the LDH lattice. Interestingly, the Ce(III)/Ce(IV) ratios were found to increase with the Ce loading amount up to 7.5% and then decrease with the Ce loading amount to 11%. The incorporation of cerium centers into LDHs was accompanied by the formation of surface/subsurface defects, *e.g*., tetrahedrally coordinated aluminum centers. As a result of cerium incorporation, the Lewis acidity of LDHs was greatly increased in parallel with slight changes in the basic properties of LDHs. The as-prepared and subsequently heat-treated (*T* = 200 °C) MgAlCe_0.075_ showed remarkable catalytic performance in the selective MPV reduction of cyclohexanone (45% cyclohexanol yield) under the optimized and mild reaction conditions (*m*(catalyst) = 100 mg; *V*(IPA) = 3 mL; *T* = 65 °C; *t* = 3 h). The catalytic activity of these systems was strongly dependent on the amount of Ce(III) centers as well as Ce(IV) centers. Moreover, the crucial role of the hydroxyl group on the surface was demonstrated, and a strongly enhanced catalytic activity of these structures was observed in the presence of surface or subsurface defect sites (such as tetrahedral aluminum cations), indicating solid FLP-like catalytic behavior. Heterogeneity, recyclability, and versatility of these systems were demonstrated, further showing the high activity of MgAlCe_0.075_ in the reduction of chosen, biomass-derived aldehydes and ketones, with the catalytic performance comparable or even superior to the benchmark catalysts.

## Supporting Information Available

Detailed experimental part; representative BET isotherms; representative SEM images, ATR-FTIR spectrum of carbonate modified structure, XRD patterns of the heat-treated and nonphase pure structures, representative TG/DTG curves, O_2_-/CO_2_-/NH_3_-TPD curves, optimization procedure of MPV reaction of aldehydes and acetophenone, and XP and FTIR spectra of the modified structures and the control experiments (PDF)
