# Supplementary material for: Isomorphic Insertion of Ce(III)/Ce(IV) Centers into Layered Double Hydroxide as a Heterogeneous Multifunctional Catalyst for Efficient Meerwein–Ponndorf–Verley Reduction
Source: ACS Appl Mater Interfaces. 2024 Feb 26;16(9):11453–66. doi: 10.1021/acsami.3c16732 (PMC10921384; doi:10.1021/acsami.3c16732)
Supplement: Supplementary file 1 — am3c16732_si_001.pdf [file am3c16732_si_001.pdf]

Supporting information

Isomorphic insertion of Ce(III)/Ce(IV) centers into layered double hydroxide as a heterogeneous multifunctional catalyst for efficient Meerwein−Ponndorf−Verley reduction

Gábor Varga,[a,b]* Thanh Truc Nguyen,[a] Jing Wang,[c] Dihua Tian,[a] Run Zhang,[a] Li Li[a] and Zhi Ping Xu[a]*

[a]*Australian Institute for Bioengineering and Nanotechnology, The University of Queensland, St. Lucia, Queensland 4072, Australia*
[b]*University of Szeged, Interdisciplinary Excellence Centre, Department of Applied and Environmental Chemistry, H-6720, Rerrich Béla tér 1, Szeged, Hungary*
[c]*Key Laboratory of OptoElectronic Science and Technology for Medicine of Ministry of Education, Fujian Provincial Key Laboratory of Photonics Technology, Fujian Normal University, Fuzhou 350117, China*

*Corresponding authors: **G. Varga** (gabor.varga5@chem.u-szeged.hu); **Z.P. Xu** (gordonxu@uq.edu.au)

Supporting information

EXPERIMENTAL PART

Materials

$MgCl_2 \times 6H_2O$, $AlCl_3 \times 6H_2O$, Na_2CO_3 and NaOH were purchased from Chem-Supply. $CeCl_3 \times 7H_2O$, cyclohexanone, cyclohexanol, methanol (MeOH), 2-propanol (2-PrOH), 2-butanol (2-BuOH), ethanol (EtOH), pyridine, CeO_2, CeO_2 nanopowder (particle size < 25nm), ZrO_2, $Zr(OH)_4$, MgO, cinnamaldehyde, cinnamyl alcohol, furaldehyde, furfuryl alcohol, crotonaldehyde, crotyl alcohol, benzaldehyde, benzyl alcohol, 1,2-hexanediol, diphenylmethanol, acetophenone, 1-phenylethanol, aniline, toluene, hexane, dimethyl sulfoxide-d6 (DMSO-d6), sodium sulfite (Na_2SO_3), 30 V/V% HCl solution and cc. HNO_3 were purchased from Sigma-Aldrich and used without any further purification. Milli-Q water was used throughout the experiments.

Preparation of hydrotalcites and their derivatives; preparation of hydrous ZrO_2

Ce-containing magnesium-aluminum layered double hydroxides (hydrotalcites, LDHs) were synthesized by a simple co-precipitation method (named in the text as one-step procedure). $MgCl_2 \times 6H_2O$ (3 mmol), (1-x) mmol $AlCl_3 \times 6H_2O$, and x mmol $CeCl_3 \times 7H_2O$ (x = 0.01–0.15 mmol) were first dissolved in 15 ml water. This mother liquor was then quickly added to 20 ml of freshly prepared NaOH solution of 0.40 M under N_2 atmosphere. After vigorous stirring for 45 min, the slurry obtained was centrifuged (4750 rpm for 5 min), washed twice with water and dispersed again (in 20 ml of water) and centrifuged (4750 rpm at 4°C for 15 min after the first step and for 30 min after the second step). The prepared gel-like product was then thoroughly redispersed in 30 ml of water and stored at room temperature for 3 days. Thereafter, the solidified final product was separated by centrifugation (4750 rpm at 4°C for 15 min) and then dried at 75°C in vacuo for 16 h. The LDHs obtained are labeled as $MgAlCe_x$, where x is the initial Ce(III) : Al(III) molar ratio: $MgAlCe_{0.01}$ – $MgAlCe_{0.15}$. Pure hydrotalcite (Mg_3Al-LDH; denoted as MgAl) was prepared in the same method in the absence of cerium salt, using 10 ml of mother liquor and 20 ml of 0.4 M NaOH solution.

The one-step method described above was used to synthesize phase-pure products, but the method was severely limited in terms of the amount of cerium centers. To overcome this limitation, a second, two-step preparation method was introduced. In this method, exactly the same reaction steps as described above were repeated until the final suspension was obtained. In this case, before the final step (separation/drying), the entire batch of cerium-containing slurry suspended in water (30 ml) was placed in a Teflon-lined stainless-steel autoclave with a

capacity of 50 ml and then heat-treated at 110°C for 16 h, followed by the final separation procedure. This method allowed the incorporation of cerium in a slightly extended concentration range (up to a cerium content of 15% compared to the Al(III) centers).

During the FT-IR study (see below), a sodium carbonate treated $MgAlCe_{0.05}$ structure was synthesized and analyzed for comparison. For this purpose, a portion (0.2 g) of the hydrotalcite thus prepared was suspended in a 0.1 M sodium carbonate solution and then stirred at room temperature for 2 h. The solid product was separated by centrifugation and then dried at 60°C for 2 hours.

To investigate the role of hydroxyl groups and water content of LDHs during the catalytic reactions, a subsequent heat treatment was carried out leading to the formation of the partially or completely dehydrated/decarboxylated LDHs/mixed oxide. For this purpose, in each case, a 3-h heat treatment was carried out in a tube furnace under N_2 protection. The temperatures applied were the following: 110, 125, 150, 175, 200, 250, 300, 400, and 500°C.

For comparison for the Raman study, both the physical mixture of CeO_2 and MgAl (CeO_2–MgAl-LDH mixture) and MgAl-LDH supported CeO_2 (CeO_2–MgAl-LDH composite) were synthesized. For the first product, CeO_2 and freshly prepared MgAl were combined in a mortar at a molar ratio of 1:20. The product was molded for 15 min using physical force. A wet impregnation method was used for the composite material. First, colloidal suspensions of CeO_2 in ethanol (5 mg/ml) were prepared. The desired amount of solution (CeO_2 from 5–15 wt%) were added to a portion of MgAl (0.1 g) and the mixture was sonicated at room temperature for 2 h. The obtained gray intermediate was separated by filtration, washed thoroughly several times with ethanol and water, and dried overnight in an oven at 60 °C. This solid was then heat-treated at 200 °C for 8 h to obtain the products.

Hydrous ZrO_2 was used in a comparative study to show the catalytic performance of some benchmark catalysts. This was prepared by a simple procedure of partial dehydration of $Zr(OH)_4$ at 120°C for 12 h in air.

Preparation of CeO_2 derivatives for mechanistic control experiments

Hydrate nanoceria was produced with precipitation method using the cerium (+3) nitrate hexahydrate ($Ce(NO_3)_3 \times 6H_2O$, purity 99.5%) dissolved in a volume of distilled water. The nitrate solution was mixed and stirred for 2 h at room temperature. Ammonium hydroxide (NH_4OH) of 25% was added to the solution in order to adjust pH (pH=10). At this step, a light-

brown precipitate was formed in the solution. This precipitate was filtered, washed with distilled water in order to remove residual NH_4^+. This initial phase was subjected to drying process at 80 °C for 10 h to deliver the partially hydrated nanoceria sample. Finally, this precursor powder was thermally treated for 6 h at 600 °C under air to give the final product.

Solid FLP-like CeO_2 was prepared by a two-step hydrothermal process. Initially, 5 mL of 0.8 M $Ce(NO_3)_3$ solution was added into 75 mL of 6.4 M NaOH aqueous solution in a 100 mL Pyrex bottle. After a 30 min reaction, the mixture was aged at room temperature for 1 h. Then, the mixture in the Pyrex bottle was reacted for 24 h at 100 °C. After cooling to room temperature, the precipitates were washed with deionized water and ethanol alternatively for three times. After drying at 60 °C, the 2 mg mL−1 of precursor solution was prepared and treated hydrothermally at 180 °C for 12 h. Finally, the product was collected by centrifugation and dried at 60°C overnight.

Characterization methods

Powder X-ray diffraction (XRD) patterns of the solids were recorded on a Bruker D8 Advance powder XRD instrument by applying CuKα radiation (λ = 0.15418 nm) and 40 kV accelerating voltage at 40 mA in the range of 2θ=5–80°. The characteristic reflections in the normalized diffractograms were identified on the basis of JCPDS-ICDD (Joint Committee of Powder Diffraction Standards- International Centre for Diffraction Data) database.

Dynamic light scattering (DLS) was used to measure the hydrodynamic size of the dispersed particles. The measurements were carried out with the same Nanosizer (Malvern) device as above at 175° scattering angle in disposable plastic cuvettes (VWR). Electrophoretic mobility was measured with the same device equipped with a 40 mW laser source operating at 658 nm wavelength. Disposable plastic omega-shaped capillary cells (Anton Paar) were used for the measurements. The obtained mobilities were then converted to zeta potentials.

The thermal behavior of the as-prepared layered composites was studied on a TGA/DSC 1 STAR^e System (Mettler-Toledo Ltd., AU). The instrument worked under constant air flow, and the heating rate was 1 °C/min. The samples, between 30 and 35 mg, were placed into high-purity alpha-alumina crucibles.

The amount of Ce, Mg and Al components in the nanoparticles was determined by inductively coupled plasma-atomic emission spectrometry (ICP-AES) using a Varian Vista Pro instrument. Before measurements, few milligrams of the samples measured by analytical accuracy were dissolved in 1.0 mL of concentrated nitric acid in 8 hours, and then, they were

diluted with distilled water to 50 mL and then filtered. Ce(III)-content and the Ce(III):Ce(IV) actual molar ratios were determined by fluorescence spectroscopy (FLS) measurements using a SHIMADZU RF-5301PC spectrometer with excitation and emission slits of 5 nm. Prior to the measurements, one portion of the as-prepared hydrotalcites were completely dissolved in cc. HCl and then diluted to the appropriate concentration. 1-cm quartz cells were used for all measurement. The measurements were carried out at room temperature upon using λ_{ex} = 255 nm and λ_{em} = 355 nm. For determining the actual molar ratios, during the preparation procedure, sodium sulfite in excess to the theoretical maximum of cerium-content was added into the solution of LDHs to reduce Ce(IV) centers, which do not have fluoresce activity, to Ce(III).

Nitrogen sorption isotherms of samples were obtained using Quantachrome Autosorb-1 analyzer at 77 K. Prior to the measurement, the samples were de-gassed at 120 °C for at least 3 h. The Brunauer-Emmett-Teller (BET) specific surface areas were calculated using adsorption data at a relative pressure range of p/p_0 = 0.05–0.25. The morphologies of the samples prepared were studied by scanning electron microscopy (SEM). The SEM images were recorded on an FEI Quanta 650 FEG at an acceleration voltage of 20.0 kV.

The instrument for taking the Fourier-transform infrared (FT-IR) spectra was a Nicolet 5700 FT-IR spectrometer (Thermo Electron Corporation) with 2 cm^{-1} resolution in ATR mode (ATR-FT-IR). The 4000–600 cm^{-1} wavenumber ranges were recorded, averaging 256 scans for each spectrum. During the adsorption measurements for analyzing the surface acidity/basicity of the solids, the same FT-IR equipment was used for recording the corresponding spectra. The surface acidity of LDHs was determined by adsorption of pyridine on self-supporting wafers (10 mg/cm^2). FT-IR spectra of adsorbed samples were recorded in the range 1700–1400 cm^{-1}. The self-supporting wafer was placed between two CaF_2 windows. Before the adsorption, the wafers were degassed at 130°C for 2 h at 10^{-4} N/m^2. The pyridine was then adsorbed on the surface of the samples at room temperature for 1 hour followed by outgassing at room temperature for 2h at 10^{-4} N/m^2. The FT-IR measurements were carried out at room temperature. To correctly interpret the results, the adsorption-desorption procedure was repeated on using outgas temperature of 125°C after adsorption. The determination of the surface basicity of LDHs was performed by adsorption of methanol in a similar way as it was above mentioned for pyridine adsorption. A minor change was that a lower outgas temperature (T = 45°C) was used during the desorption.

Raman spectra were recorded with a portable IM-52 Raman Microscope (Snowy Range Instruments). The 785 nm laser wavelength with a laser power of 70 mW was used for excitation

of Raman scattering. Raman spectra were obtained at 10 s integration time using a 50X microscope objective.

The first coordination sphere and oxidation state of the transition metal ions was established by using an X-ray photoelectron (XP) spectroscopic mapping. X-ray photoelectron spectra (XPS) were recorded with a SPECS instrument equipped with a Kratos Axis Supra Plus XPS, under a main-chamber pressure in the 10^{-9}–10^{-10} mbar range. The analyzer was run in the fixed analyzer transmission (FAT) mode with 20 eV pass energy. The Al Kα radiation (hν = 1486.6 eV) of a dual anode X-ray gun was applied as an excitation source. The gun was operated at 210 W power (14 kV, 15 mA). The binding energy scale was corrected by setting the main C1s component to 284.1 eV in all cases. ^{27}Al (cross-polarization magnetic angle spinning) solid state-(SS)-NMR measurements were carried out on a Bruker Avance III spectrometer with a 300 MHz magnet equipped with a 4 mm double air bearing, magic angle spinning probe. During the measurements, a zirconia rotor with a Kel-F cap was used. The samples were rotated at 5 kHz.

$O_2/NH_3/CO_2$–TPD (temperature programmed desorption) was conducted using a BELCAT-A apparatus with a thermal conductivity detector (TCD) to assess the surface acidity and basicity of catalysts, respectively. The sample was pretreated under a He flows at 300°C for 1 h. Subsequently, the sample was cooled to 50°C, and subjected to a mixture of $O_2/NH_3/CO_2$–He gas flow for 1 h, followed by flushing with pure He gas to remove physically adsorbed $O_2/NH_3/CO_2$. Finally, TPD data were collected from 50°C to 600°C.

Supporting information

Cyclohexanone transfer hydrogenation to cyclohexanol in 2-PrOH

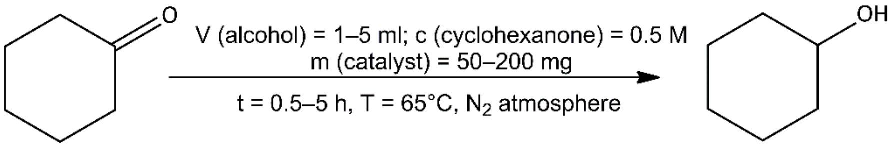

Scheme S1. The applied cyclohexanone-to-cyclohexanol transfer hydrogenation test reaction. (alcohol: EtOH, 2-propanol, 2-butanol)

The Meerwein–Ponndorf–Verley(MPV) reactions of cyclohexanone, which were used as test reactions to describe the catalytic performance of the as-prepared solids, were carried out in a batch reactor at a constant reaction temperature of 65°C under a N_2 atmosphere. A solution of cyclohexanone (c = 0.5 M) and the appropriate amount of 2-PrOH (2–4 ml) was stirred under the above mentioned reaction conditions in the presence of a chosen hydrotalcite derivative (50–200 mg) for an appropriate reaction time (1–360 min). When the reaction was completed, the obtained slurry was cooled down to room temperature and then centrifuged at 10,000 rpm for 10 min to remove the catalyst. The obtained mixture was then evaporated under reduced pressure and re-dissolved in 2-PrOH. The reaction parameters were optimized by Box-Behnken design (BBD). The chosen reaction parameters can be seen in Table S1. (X_1: Catalyst loading; X_2: Quality of H-source; X_3: Quantity of solvent (H-source))

Table S1. Coded factor levels for a Box-Behnken design of a three-variable system.

	**X_1**	**X_2**	**X_3**
**–1**	50 mg	EtOH	1 ml
**0**	100 mg	2-PrOH	3 ml
**1**	200 mg	2-BuOH	5 ml

**Experience**	**X_1**	**X_2**	**X_3**
**1.**	−1	−1	0
**2.**	1	−1	0
**3.**	−1	1	0
**4.**	1	1	0
**5.**	−1	0	−1
**6.**	1	0	−1
**7.**	−1	0	1
**8.**	1	0	1
**9.**	0	−1	−1
**10.**	0	1	−1
**11.**	0	−1	1
**12.**	0	1	1
**13.**	0	0	0

During the recycling tests, after the reductive transformations, the active catalyst was separated from the reaction mixture by centrifugation followed by thorough washing with ethanol and water. After that, the catalyst was re-activated at 200°C and then reutilized in the

following runs under the optimized reaction conditions. To determine the structural integrity of the composites after each run, *ex-situ* XRD study on the spent catalysts was performed. To ascertain the heterogeneous nature of the reactions, the hot filtration test was carried out as follows. The catalytic composite was filtrated from the reaction slurry before completion of the transformation and then the filtrate was further treated under unchanged reaction conditions.

Cyclohexanone conversions were determined by ultraviolet-visible (UV-Vis) spectrophotometry in 2-PrOH on using the absorption band maximum of the cyclohexanone in the UV region (λ_{max} = 282 nm) associated with the n → π* transition (Figure S1, left). The UV-Vis absorbances were detected in a SHIMADZU UV-2450 spectrophotometer. For determining the cyclohexanol yields, ^{1}H-NMR measurements were introduced (Figure S1B,C). ^{1}H-NMR spectra were recorded at room temperature on a Bruker AV-500 in DMSO-d6. The actual cyclohexanol yield could be calculated by determining the actual ratio of the integrated peak areas (Figure S1C) of –OH proton shift (Figure S1B/orange, 4.41 ppm) from cyclohexanol and α–CH_2 protons shift from cyclohexanone (Figure S1B/light blue, 2.26 ppm).

To present the versatility of the catalysts, a short scope of the catalytic reaction was also introduced. During these tests, transfer hydrogenation of cinnamaldehyde, crotonaldehyde, furaldehyde, benzaldehyde and acetophenone was investigated after a shortened optimization procedure. These were implemented as the transfer hydrogenation of cyclohexanone. During scope, the catalytic markers were determined *via* the same method as above presented applying the below listed NMR signals and UV-Vis absorbances (Table S2).

CONTROL EXPERIMENTS

N-Alkylation of aniline with alcohols (benzyl alcohol, 1,2-hexanediol and diphenylmethanol) was carried out in a batch reactor. Typically, aniline (0.75 mmol), the corresponding alcohol (0.5 mmol) and the corresponding catalyst (100 mg) were added successively to toluene of 5ml. Then the reaction mixture was stirred (600 rpm) at 60 °C under air or an N_2 atmosphere for 16 hours. Thereafter, the reaction vessel was immediately cooled down in ice water. The reaction mixture was analysed using a Hewlett–Packard 5890 Series II gas chromatograph (GC) equipped with flame ionization detector, using an Agilent HP-5 column and the internal standard (hexane) technique. Alcohol oxidation/dehydrogenation reactions were carried out in the same way, without adding any acceptor molecule (*e.g.* aniline) to the system.

Supporting information

STATISTICAL ANALYSIS

All experiments were conducted at least in triplicate with the data expressed as mean ± standard error of the mean. The student t-test was used to test the significant difference between the experimental groups. NS: no significant difference when $p > 0.05$; *: $p < 0.05$; **: $p < 0.01$; ***: $p < 0.001$; and ****: $p < 0.0001$.

Supporting information

Table S2. Fingerprint-like NMR shifts and UV-Vis transition states used for following the catalytic reactions during scope.

Reactant	Fingerprint NMR shift (ppm)[a]	$n \rightarrow \pi^*$ transition (nm)[b]	Product	Fingerprint NMR shift (ppm)[a]
**Cinnamaldehyde**	9.80 (–CHO)	298	Cinnamyl alcohol	2.05 (–OH)
**Crotonaldehyde**	9.60 (–CHO)	329	Crotyl alcohol	1.97 (–OH)
**Furaldehyde**	9.90 (–CHO)	284	Furfuryl alcohol	2.70 (–OH)
**Benzaldehyde**	10.10 (–CHO)	256 ($\pi \rightarrow \pi^*$)	Benzyl alcohol	4.70 ($–CH_2$)
**Acetophenone**	2.70 ($–CH_3$)	252 ($\pi \rightarrow \pi^*$)	1-phenylethanol	1.60 (–OH)

a: ^{1}H-NMR spectra, detected in DMSO-d6; b: detected by UV-Vis spectroscopy using 2-PrOH solvent.

Supporting information

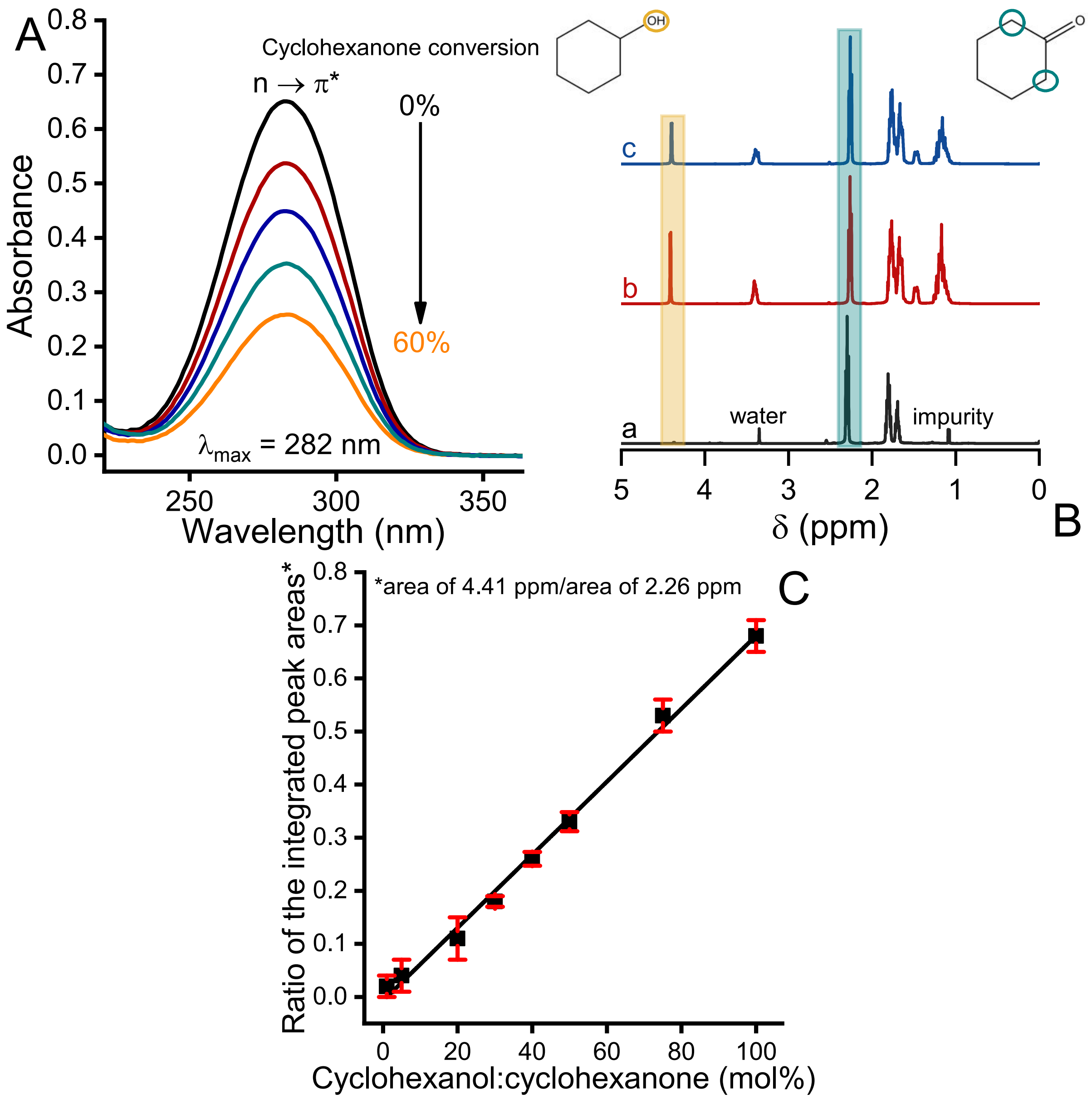

Figure S1. (A) UV-Vis spectrum of variable amounts of cyclohexanone in the wavelength region of 240–360 nm. (B) ^{1}H-NMR spectrum of (a) cyclohexanone after heating in 2-PrOH at 65°C for 5 in the absence of any catalyst under a N_2 atmosphere; (b) cyclohexanone : cyclohexanol mixture of 1:1 molar ratio (equal to 50mol% cyclohexanone conversion with 50 mol% cyclohexanol yield); (c) reaction mixture after catalytic reaction of cyclohexanone (0.5M) in 2-PrOH of 3 ml in the presence of $MgAlCe_{0.075}$ catalyst (pre-treated at 175°C) at 65°C for 3h under a N_2 atmosphere (cyclohexanol yield of 39% with 100% cyclohexanol selectivity). (C) Calibration curve for determining the actual cyclohexanol yield. Ratio of the integrated peak areas of 4.41 ppm and 2.26 ppm chemical shifts as a function of molar ratio of cyclohexanol: cyclohexanone in the calibration line.

Supporting information

SUPPORTING RESULTS

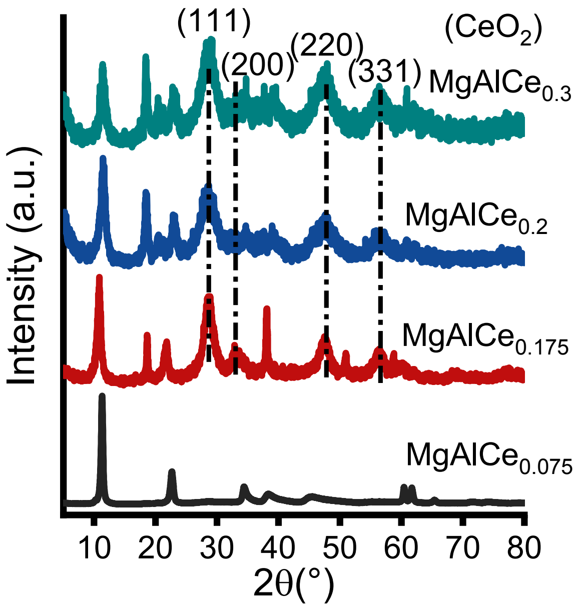

Figure S2. XRD patterns of as-prepared hydrotalcites with different Ce(III):Al(III) initial ratios synthesized *via* a one-step procedure.

Supporting information

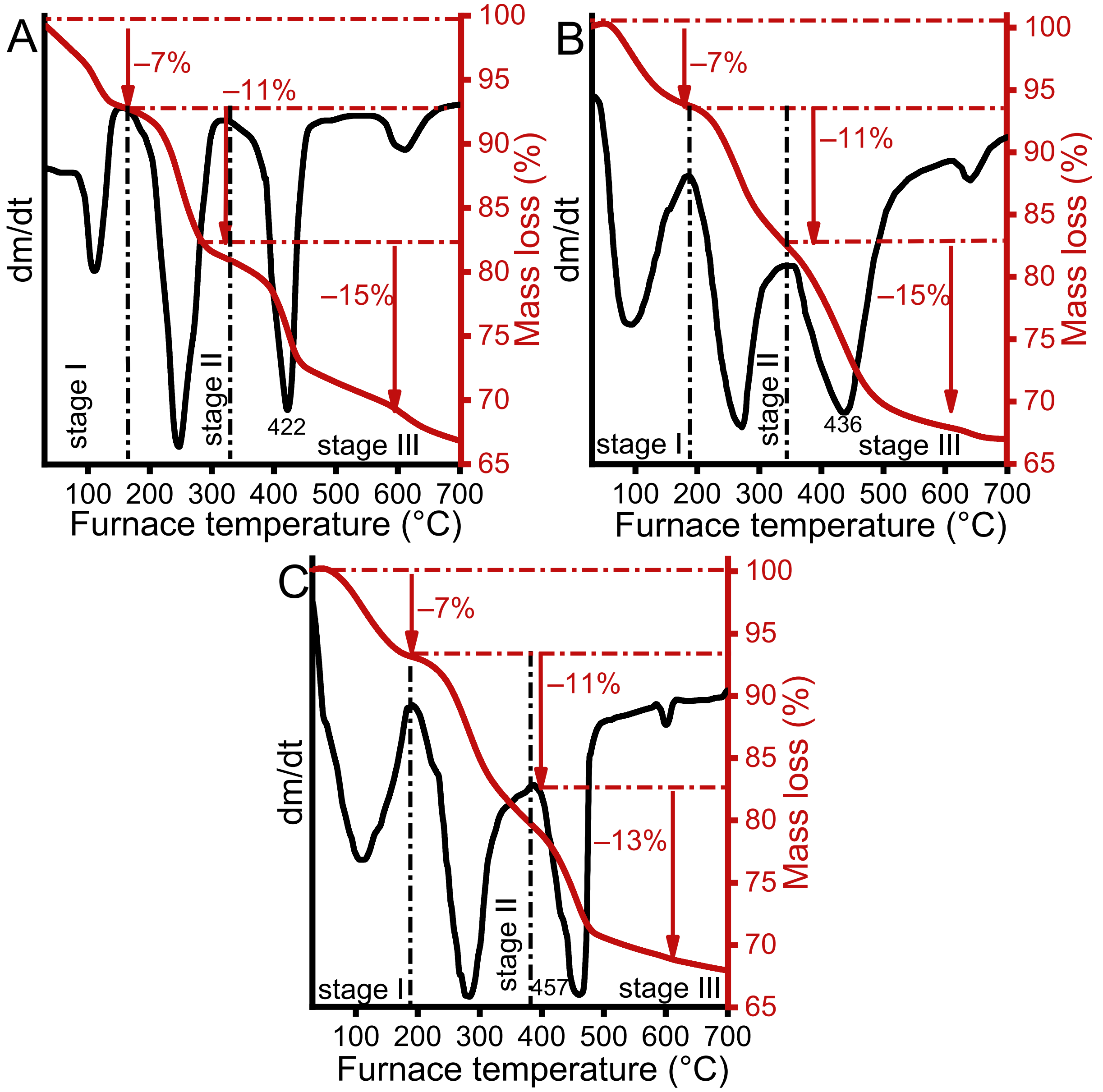

Figure S3. Representative TG/DTG curves of the as-prepared hydrotalcites. (A) MgAl; (B) $MgAlCe_{0.075}$; (C) $MgAlCe_{0.15}$.

Supporting information

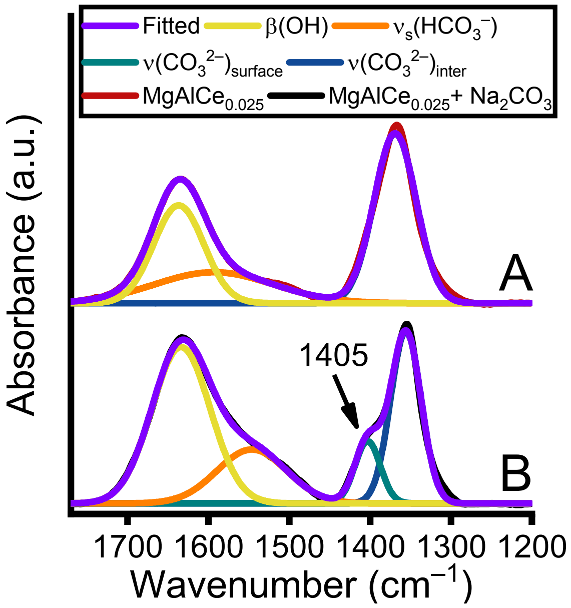

Figure S4. ATR-FT-IR spectra of (A) as-prepared $MgAlCe_{0.05}$ and (B) $MgAlCe_{0.05}$ after post synthetic treatment in Na_2CO_3.

Supporting information

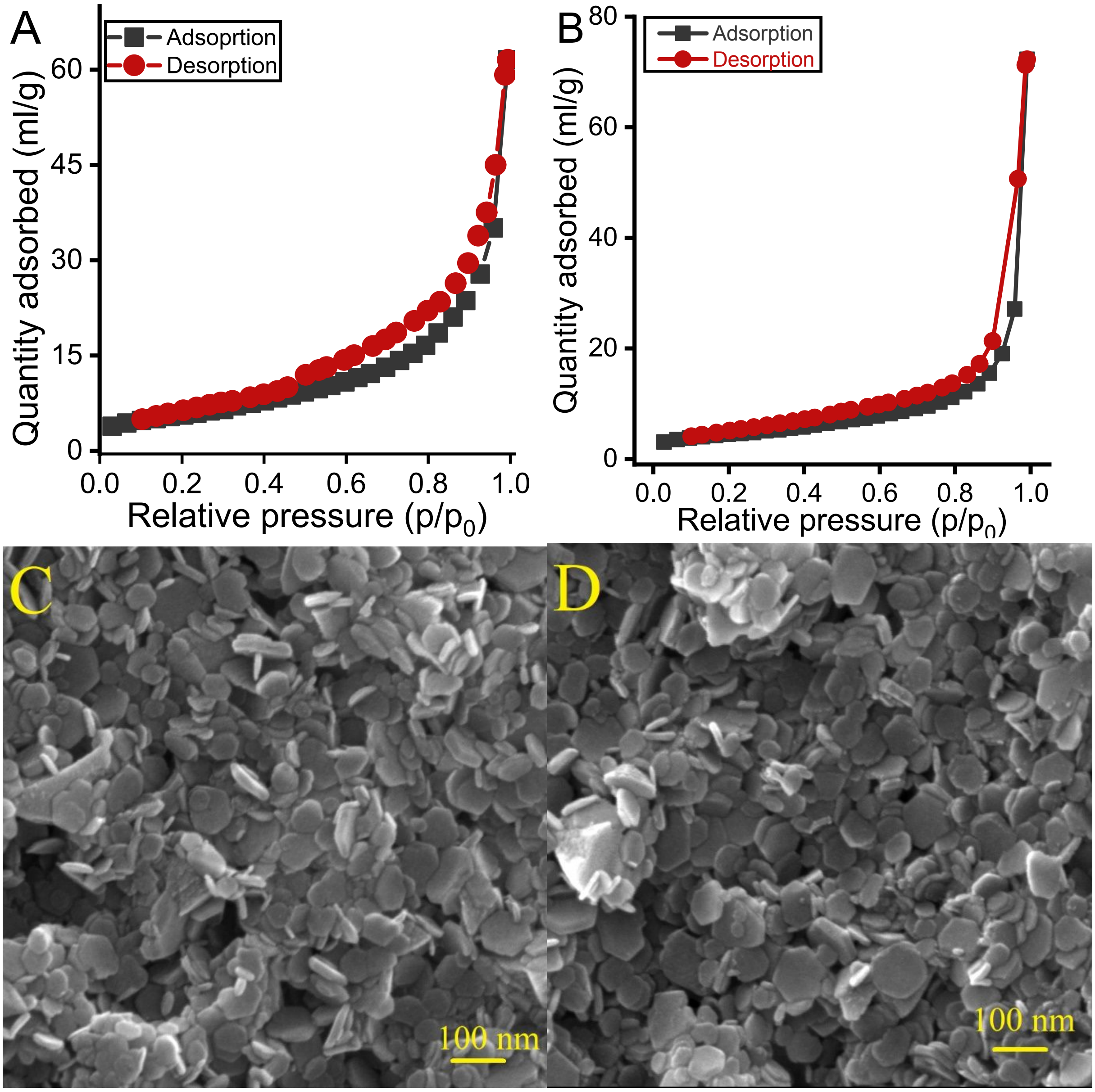

Figure S5. Representative N_2 sorption isotherm of the as-prepared hydrotalcites: (A) MgAl; (B)$MgAlCe_{0.075}$. Representative SEM images of the as-prepared hydrotalcites: (C) MgAl; (D) $MgAlCe_{0.075}$.

Supporting information

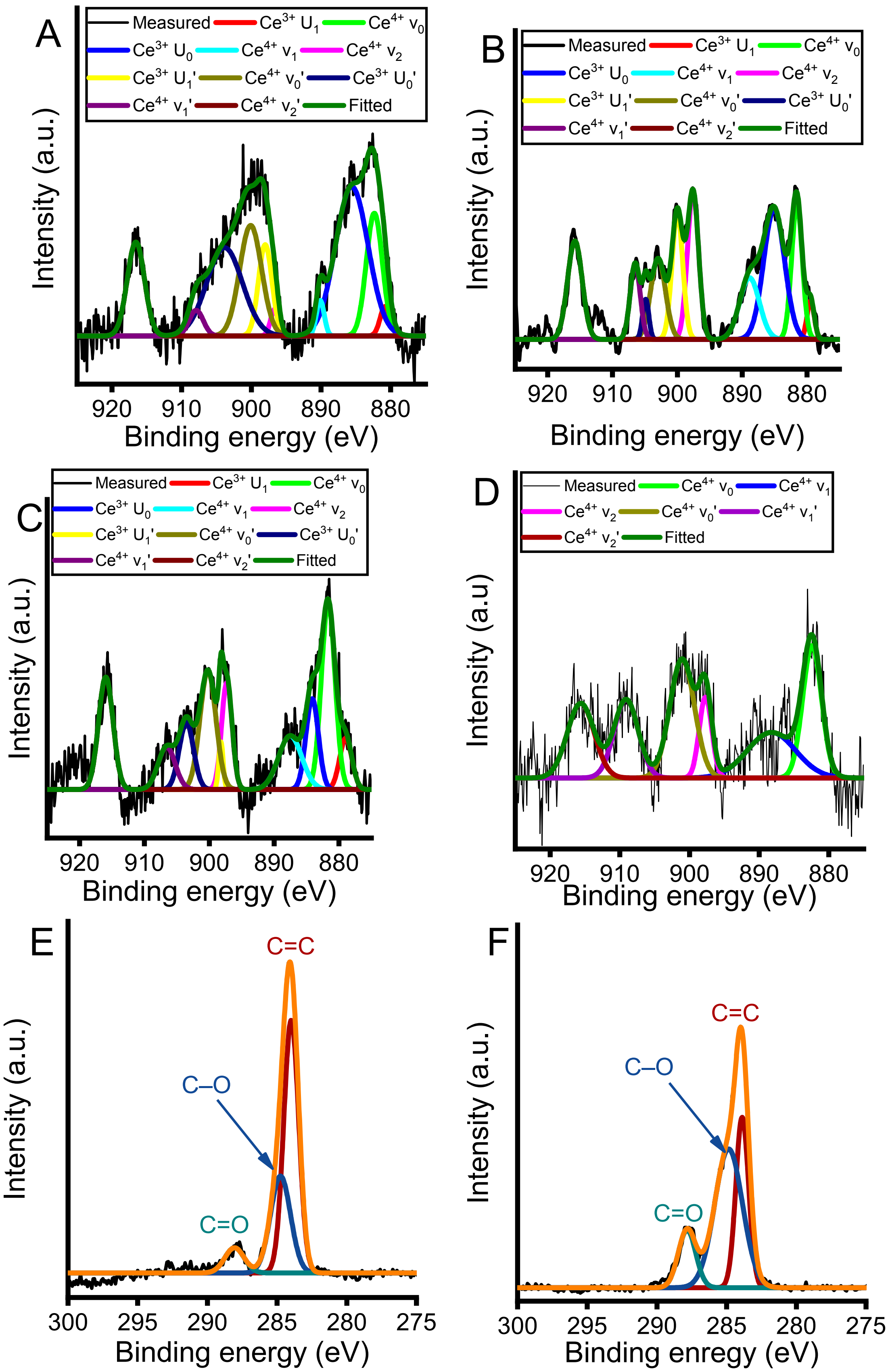

Figure S6. Ce3d XP spectra of (A) $MgAlCe_{0.025}$; (B) $MgAlCe_{0.05}$; (C) $MgAlCe_{0.1}$ and (D) $MgAlCe_{0.125}$. Representative C1s XP spectra of (E) MgAl and (F) $MgAlCe_{0.075}$.

Supporting information

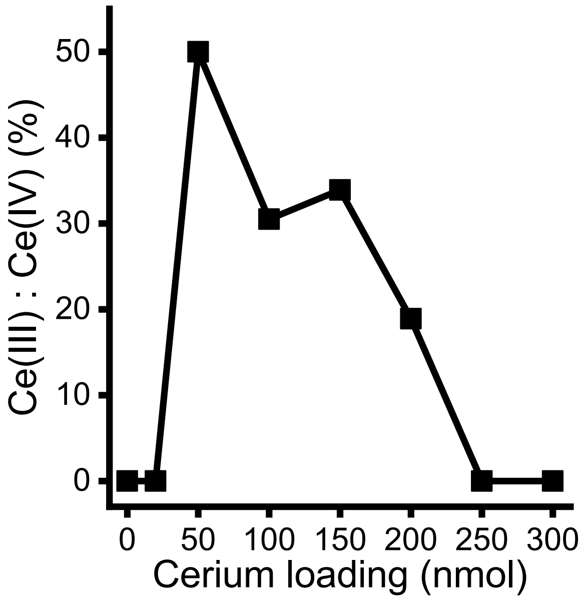

Figure S7. Ce(III)-to-Ce(IV) actual molar ratios in the as-prepared samples calculated from Ce3d XPS results as a function of the initial cerium loading.

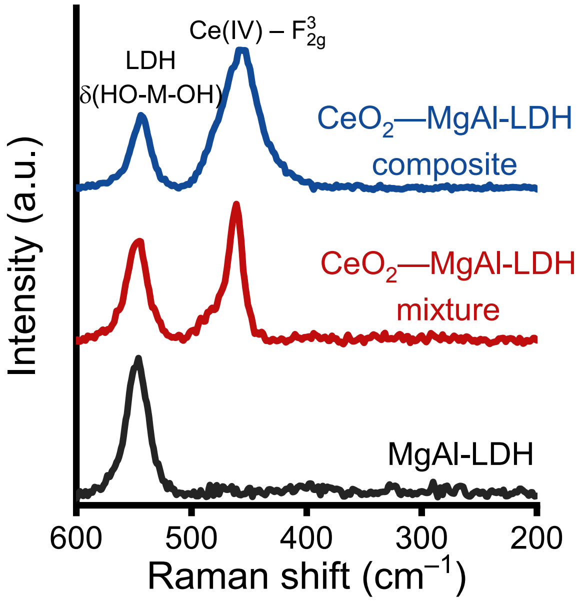

Figure S8. Raman spectra of non-modified hydrotalcite, CeO_2–MgAl-LDH physical mixture (mixture) and MgAl-LDH supported CeO_2 (composite). (Latter two were synthesized for a comparison.)

Supporting information

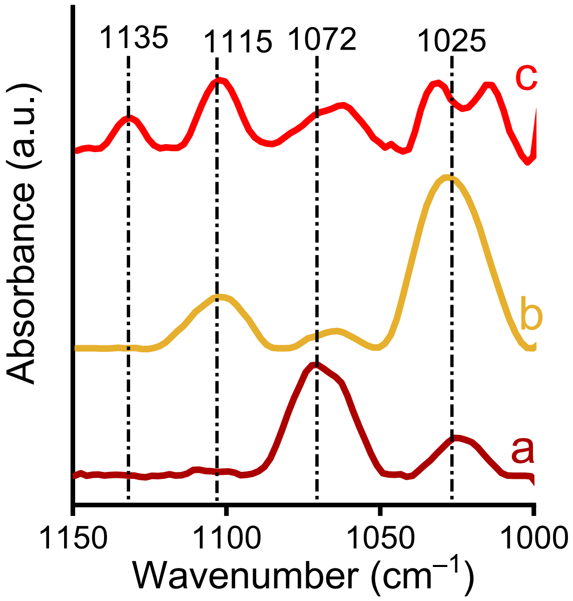

Figure S9. ATR-FT-IR spectra of (a) $MgAlCe_{0.05}$; (b) $MgAlCe_{0.075}$ and (c) $MgAlCe_{0.125}$ recorded after adsorption of methanol at room temperature on the samples. (All the presented spectra are difference spectra obtained after the subtraction of the corresponding spectra of the hydrotalcites without adsorbed probe molecules.)

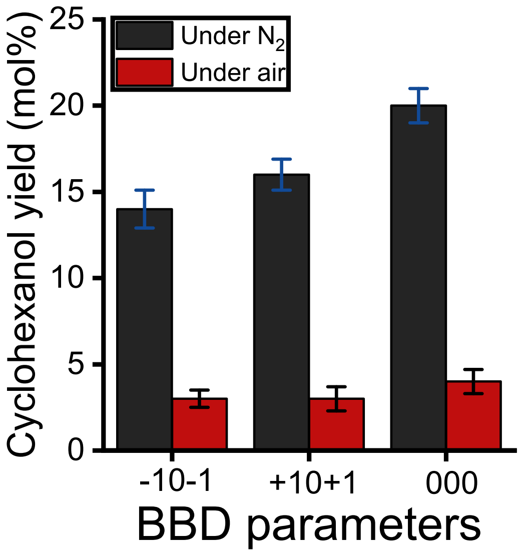

Figure S10. The impact of the air atmosphere when introducing experimental runs of Box-Benken design (BBD). (Parameter web: X1: -1 = 50 mg catalyst (MgAlCe0.05); 0 = 100 mg catalyst; 1 = 200 mg catalyst; X2: -1 = EtOH (used as H-source and solvent); 0 = 2-propanol; 1 = 2-butanol; X3: -1 = 1 ml solvent; 0 = 3 ml; 1 = 5 ml.)

Supporting information

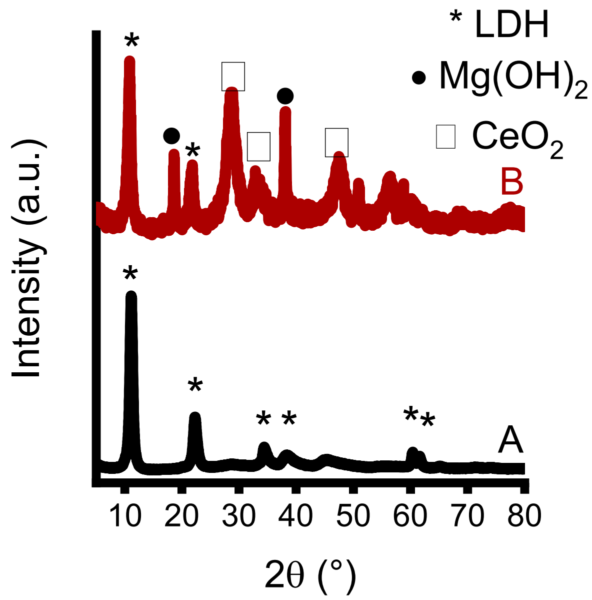

Figure S11. XRD patterns of as-prepared (A) and (B) spent $MgAlCe_{0.075}$. Reaction conditions: c (cyclohexanone) = 0.5M; V (2-propanol) = 5 ml; m (catalyst) = 200 mg; t = 5h; T = 65°C; under an air atmosphere.)

Supporting information

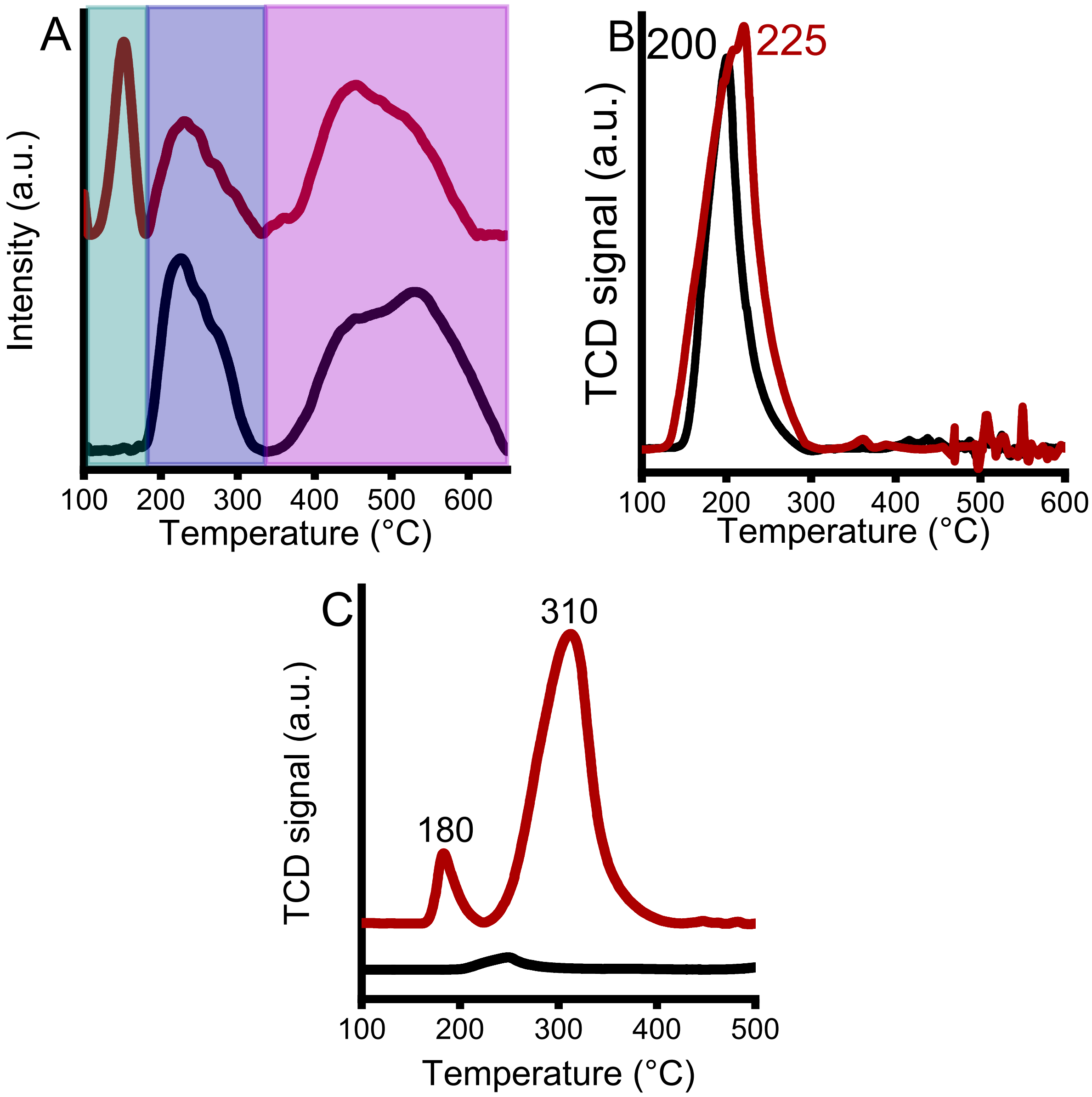

Figure S12. O_2-TPD profile (A), CO_2–TPD profile (B) and NH_3–TPD profile (C) of MgAl (black lines) and $MgAlCe_{0.075}$ (red lines).

Supporting information

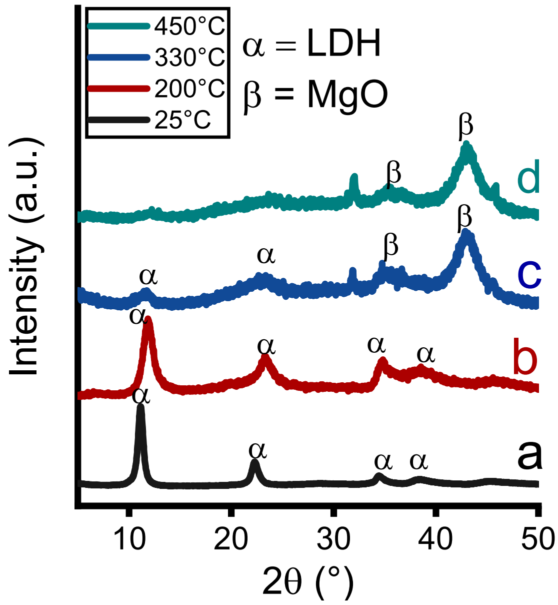

Figure S13. XRD patterns of (a) as-prepared $MgAlCe_{0.075}$; (b) $MgAlCe_{0.075}$ heat-treated at 175°C; (c) $MgAlCe_{0.075}$ heat-treated at 300°C and (d) $MgAlCe_{0.075}$ heat -treated at 400°C.

Supporting information

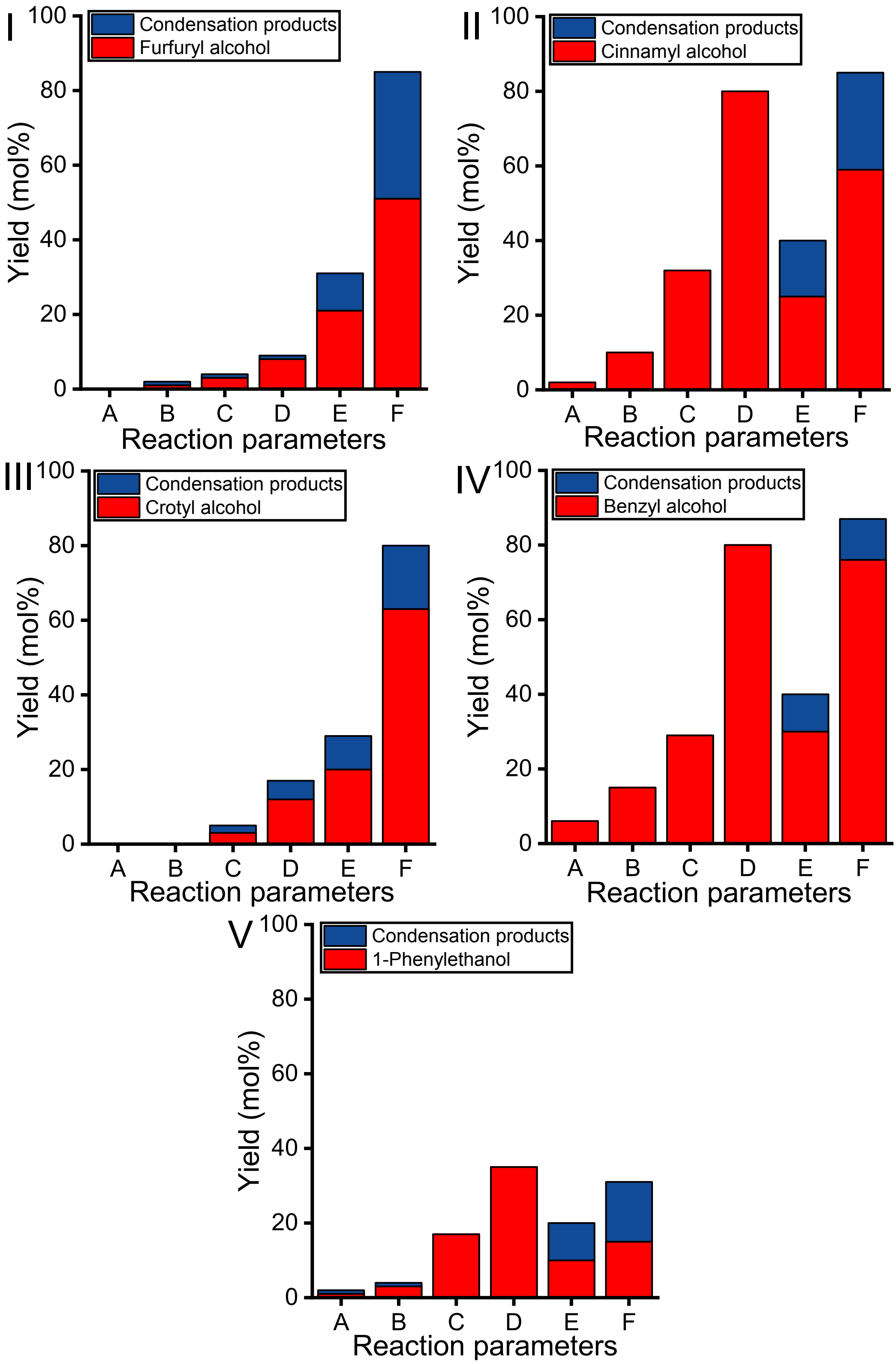

Figure S14. Shortened optimisation procedure during scope of transfer hydrogenation reaction of different aldehydes and ketones (0.5 M) in 2-PrOH of 3 ml catalysed by $MgAlCe_{0.075}$ pre-treated at 200°C of 100 mg under a N_2 atmosphere. Reactants: (I) Furaldehyde; (II) Cinnamaldehyde; (III) Crotonaldehyde; (IV) Benzaldehyde and (V) acetophenone. Reactions were carried out: A: at 65°C for 2h; B: at 65°C for 8h; (C) at 82°C for 2h; (D) at 82°C for 8h; (E) at 130°C for 2h and (F) at 130°C for 8h.

Supporting information

Table S3. BET surface area, average particle size and zeta potential of the as-prepared layered double hydrixides.

Composites	BET surface area (m^2/g)[a]	Average particle size (nm)[b]	Zeta potential (mV)[b]
**MgAl**	63±5	91±7	32 ± 1
**$MgAlCe_{0.01}$**	60±4	99±8	30 ± 2
**$MgAlCe_{0.025}$**	73±10	93±5	28 ± 1
**$MgAlCe_{0.05}$**	84±2	114±10	29 ± 3
**$MgAlCe_{0.075}$**	55±8	121±8	26 ± 2
**$MgAlCe_{0.1}$**	50±11	119±10	28 ± 2
**$MgAlCe_{0.125}$**	52±6	144±12	24 ± 2
**$MgAlCe_{0.15}$**	47±10	167±12	25 ± 4

a: determined by N_2 sorption measurements using BET calculation method; b: determined by DLS

Supporting information

Table S4. Fitted XPS parameters

Transition type	Component	Energy range (eV)
**Al2p**	**Al**–OH octahedrally coordinated	530.6–531.0
	partially dehydrated **Al**–OH (defect site)	531.8–532.2
**O1s**	Al–**O**H/Mg–**O**H	74.1
	partially dehydrated Al–**O**H (defect site)	74.8–75.4
**Ce3d**	Ce^{3+} u_0	884.9–885.1
	Ce^{3+} u_1	879.5–879.7
	Ce^{3+} u_0’	904.7–904.9
	Ce^{3+} u_1’	898.8–899.0
	Ce^{4+} v_0	881.5–882.0
	Ce^{4+} v_1	888.4–889.0
	Ce^{4+} v_2	897.6–898.0
	Ce^{4+} v_0’	901.1–902.2
	Ce^{4+} v_1’	907.0–908.0
	Ce^{4+} v_2’	915.7
**C1s**	C=C	284.1
	C–O	284.8–285.2
	C=O	287.6–287.9

Supporting information

Scheme S2. Control Experiments

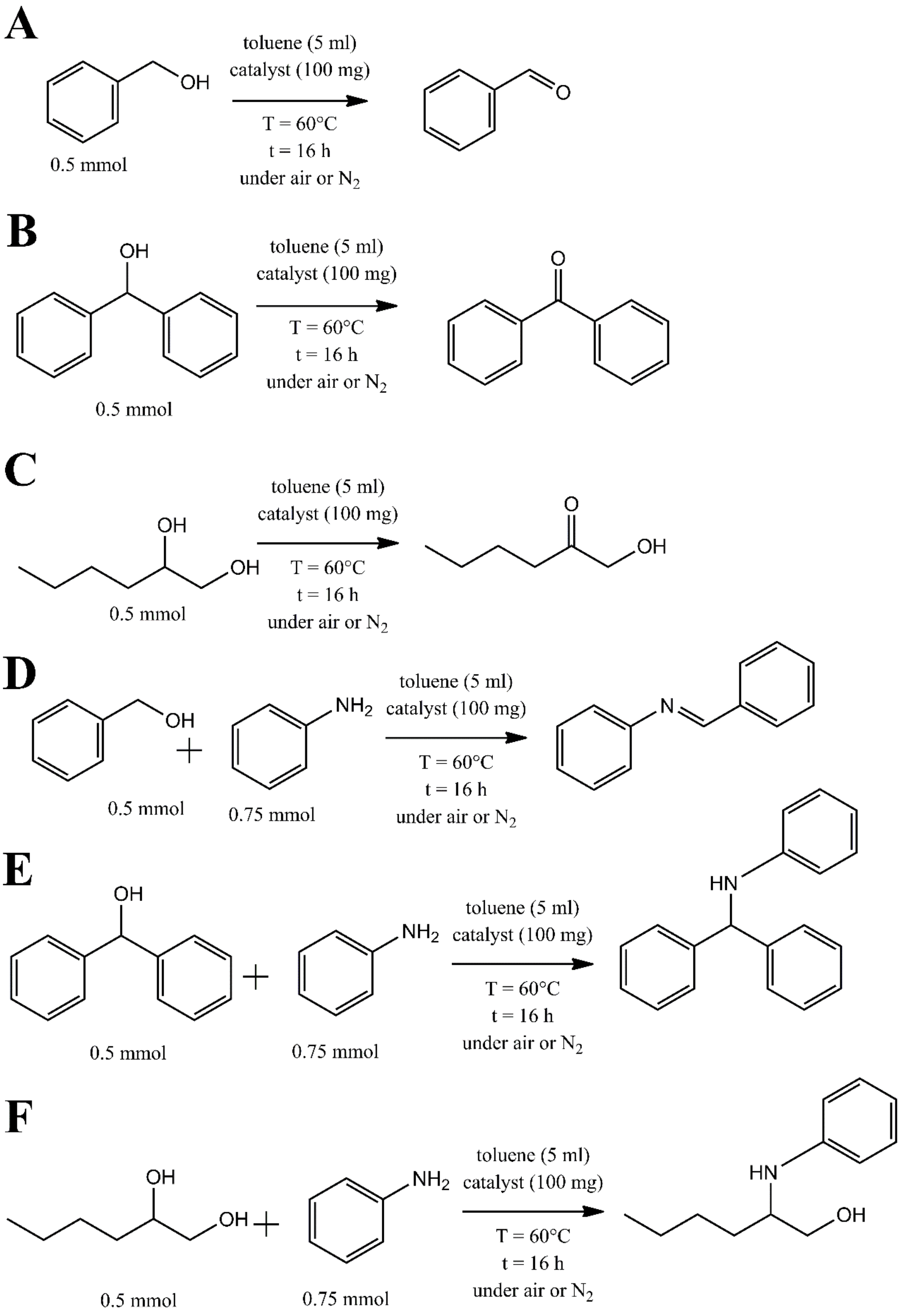

A: benzyl alcohol oxidation; B: diphenylmethanol oxidation; C: 1,2-hexanediol oxidation; D: N-alkylation of aniline with benzyl alcohol; E: N-alkylation of aniline with diphenylmethanol; F: N-alkylation of aniline with 1,2-hexanediol

Supporting information

Table S5. Results of the catalytic control experiments using different catalytic systems (Reactions are marked as in Scheme S1 (A–F))

	**Reaction**	**Conversion of alcohol (mol%) (under an air atmosphere)**							
		**LDH**	**deh. LDH**	**CeO_2 (ox.)**	**CeO_2 (red.)**	**FLP-CeO_2[a]**	**hyd. nanoceria[b]**	**$Ce(OH)_3$[c]**	**$MgAlCe_{0.075}$[d]**
**1.**	**A**	—	—	3	11	12	23	14	27
**2.**	**B**	—	—	—	4	6	11	7	16
**3.**	**C**	—	—	—	—	—	—	—	—
**Conversion of alcohol (mol%) (under a N_2 atmosphere)**									
**4.**	**A**	—	—	—	—	4	9	—	12
**5.**	**B**	—	—	—	—	—	1	—	5
**6.**	**C**	—	—	—	—	—	—	—	—
**Conversion of alcohol (mol%) (reactions with aniline under a N_2 atmosphere)**									
**7.**	**D**	—	—	—	—	10	17	11	25
**8.**	**E**	—	—	—	—	7	10	6	16
**9.**	**F**	—	—	—	—	3	6	3	10

LDH = MgAl; deh.: partially dehydrated (180°C); ox.: oxidized at 250°C; red.: reduced at 300°C; hyd.: hydrated; a: as-synthesized based on: Y. Zhou et al. "Highly selective transfer hydrogenation of furfural into furfuryl alcohol by interfacial frustrated Lewis pairs on CeO_2" *J. Catal.* **410** (2022) 54–62. doi: 10.1016/j.jcat.2022.04.010; b: as-synthesized based on: L. Bourja et al. "Structural modifications of nanostructured ceria CeO_2, x H_2O during dehydration process" *Powder Technol.* **215–216** (2012) 66–71. doi: 10.1016/j.powtec.2011.09.008; c: as-synthesized based on: R.F. Andre et al. "From $Ce(OH)_3$ to Nanoscaled CeO_2: Identification and Crystal Structure of a Cerium Oxyhydroxide Intermediate Phase" *Chem. Mater.* **35** (2023) 5040–5048. doi: 10.1021/acs.chemmater.3c00486; d: as-prepared and then heat treated at 180°C
